# Green Hydrogel Synthesis: Emphasis on Proteomics and Polymer Particle-Protein Interaction

**DOI:** 10.3390/polym14214755

**Published:** 2022-11-06

**Authors:** Liana Chafran, Amy Carfagno, Amaal Altalhi, Barney Bishop

**Affiliations:** Department of Chemistry and Biochemistry, George Mason University, Manassas, VA 20110 , USA

**Keywords:** green synthesis, hydrogel, biomaterials, proteomics, drug discovery

## Abstract

The field of drug discovery has seen significant progress in recent years. These advances drive the development of new technologies for testing compound’s effectiveness, as well as their adverse effects on organs and tissues. As an auxiliary tool for drug discovery, smart biomaterials and biopolymers produced from biodegradable monomers allow the manufacture of multifunctional polymeric devices capable of acting as biosensors, of incorporating bioactives and biomolecules, or even mimicking organs and tissues through self-association and organization between cells and biopolymers. This review discusses in detail the use of natural monomers for the synthesis of hydrogels via green routes. The physical, chemical and morphological characteristics of these polymers are described, in addition to emphasizing polymer–particle–protein interactions and their application in proteomics studies. To highlight the diversity of green synthesis methodologies and the properties of the final hydrogels, applications in the areas of drug delivery, antibody interactions, cancer therapy, imaging and biomarker analysis are also discussed, as well as the use of hydrogels for the discovery of antimicrobial and antiviral peptides with therapeutic potential.

## 1. Introduction

Hydrogels are widely used in the biomedical field as substrates for cell culture, templates for tissue engineering, and vehicles for the delivery of active substances. Due to their three-dimensional, porous and hydrophilic structures, these materials have characteristics very close to natural living tissue and are able to absorb large amounts of water and biological fluids [1]. Known as reversible or physical gels, these materials have a highly reticulate structure attributable to the presence of crosslinking agents in their composition; this facilitates the incorporation and encapsulation of a variety of molecules and substrates in their pores such as cells, proteins, peptides and active substances ascribed to high diffusion capacity [2]. Depending on the molecular interactions involved during the formation of the network (ionic forces, hydrogen bonding or hydrophobic forces), hydrogels may have their structure, as well as their viscoelasticity, solubility and porosity altered by varying environmental conditions such as pH, ionic strength, light or temperature. Furthermore, depending on the nature of the functional groups present in the polymer, hydrogels may present positively or negatively charged moieties within their structure, which facilitates not only the process of swelling upon variations in pH, but also alterations in their spatial shape when exposed to an electric field [3]. The miniaturization of hydrogels to the nano- and microscale allows the construction of adaptable materials for applications where diffusion capacity inside and outside the particles is critical. Additionally, when applied to cell-based therapies, liquid micro-lenses, cancer therapy or drug delivery applications, nanogels or microgels can improve the bioavailability of therapeutic agents and their stability against chemical and enzymatic degradation, as well as prolonging drug or gene effects in target tissue [4].

The usage of nano- or microgels in the process of capturing proteins and peptides from biological samples has become an important tool in the field of proteomics, which focuses on the study of proteins or sets of proteins that are being produced by cells, tissues or organisms using separation and identification techniques, such as chromatography, electrophoresis, mass spectrometry and bioinformatics [5]. Hydrogels are capable of effectively sequestering, through chemical and physical processes, small and low-abundance proteins and peptides in plasma which are rich in information related to physiological, metabolic and disease-associated biological states [6]. This capture process, known as size exclusion/affinity technology, combines the physical properties of the particles, such as porosity and morphology, with intermolecular interaction between functional groups dispersed in the polymeric matrix and the target substrates dispersed in the biological sample. Highly abundant large proteins (above approximately 20,000 Da) are excluded from the polymeric matrix. The application of hydrogel particles in this way replaces the more complex and labor-intensive fractionation and purification steps typically associated with proteomic analysis [7]. 

This review aims to address in detail the different types of nano- or microgels described in the literature and synthesized from green routes, as well as their physical, chemical and morphological characteristics, in addition to describing polymer–particle–protein interactions and their application to proteomics studies. Furthermore, different methodologies for the application of nano- and microgels in the areas of drug delivery, antibody interactions, cancer therapy, imaging and biomarker analysis are discussed, as well as the use of hydrogel nanoparticles for the capture of antimicrobial peptides with potential use in the treatment of bacterial, immunomodulatory and viral diseases such as malaria, diabetes, tuberculosis, HIV-1, influenza A, Zika virus and respiratory syncytial virus (RSV). Finally, potential therapeutic applications using hydrogel nanoparticles are discussed, emphasizing the importance of research into the development of new polymeric particles capable of separating and encapsulating active substances from different biological fluids.

## 2. Hydrogels

Hydrogels belong to the class of so-called polymeric gels, compounds characterized by their three-dimensional structure formed by covalent and non-covalent bonds in solution (polymer/solvent systems), with ability to reversibly swell or shrink (up to 1000 times in volume, depending on the monomer and crosslinking agents used). Hydrogels exhibit pseudoplastic (non-Newtonian) rheological behavior; in other words, they tend to show declining viscosity with an increase in shear rate. When in the presence of electrolytes or highly charged functional groups, polymeric gels are able to interact with surfactants or substrates containing functional groups with charges opposite to that of the polymeric matrix through Coulombic interactions, thereby acquiring electrostatic properties such as electrical oscillation, electrical contraction and mechanoelectrical effect [8]. In the case of hydrogels, their high absorption capacity is due to the presence of hydrophilic functional groups dispersed in the polymeric backbone that retains liquid inside the porous structure after the diffusion process. Hydrogel performance in terms of swelling or shrinking can be improved through the addition of crosslinked monomers or polymers during preparation [9]. In the field of engineering and technology, the use of hydrogels is associated with processes that require considerable mechanical strength, biocompatibility, functionality, degradability, physical integrity in aqueous media and considerable adaptability to external stimuli [10]. Owing to their ability to incorporate several functional groups such as carboxyl groups, amides, imides, amines, hydroxyls and sulfonic acid groups, among others, hydrogels can be prepared from three-dimensional polymerization, in which a monomer with hydrophilic properties polymerizes in the presence of a crosslinking agent containing more than one functional group, or by the direct crosslinking of water-soluble polymers. Both types of reactions are usually initiated by compounds capable of generating free radicals, such as potassium persulfate (KPS), 2,2-*azo*-isobutyronitrile (AIBN), ammonium peroxodisulfate (APS), and benzoyl peroxide, among others. Furthermore, ultraviolet radiation (UV), gamma radiation or electron beam radiation can also be used in order to assist the formation of radical species with the objective of achieving higher yield and a reduced generation of byproducts under mild reaction conditions [11]. 

**Figure 1:**
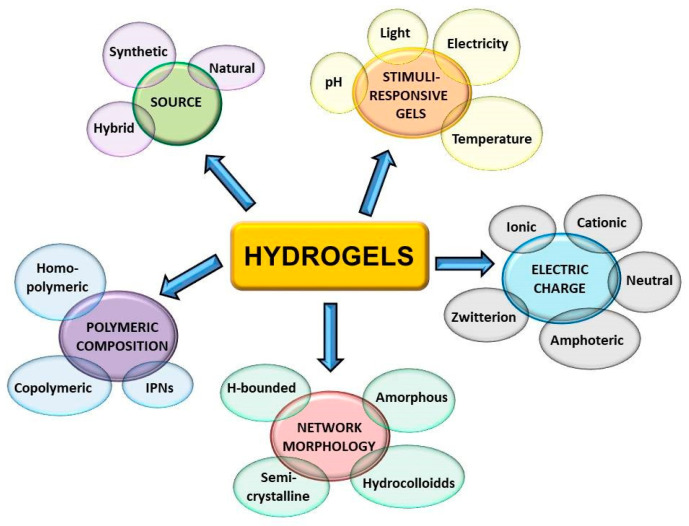
Representation of different classifications attributed to hydrogels according to the source, the polymeric composition, network morphology, electric charge and sensitivity to changes in environmental conditions (stimuli-responsive gels).

Hydrogels are classified according to their starting material (natural, synthetic or hybrid), polymeric composition (homopolymeric, copolymeric or multipolymer interpenetrating polymeric hydrogel), network morphology (amorphous, semi-crystalline, hydrogen-bonded or hydrocolloids), electrical charge (ionic, cationic, amphoteric, neural or zwitterionic), sensitivity to changes in environmental conditions (pH, temperature, electricity, light or presence of enzymes) and type of crosslinking system used (physical, chemical or biochemical) [12]. The different classifications used to describe hydrogels are summarized in Figure 1.

Within the context of green chemistry, in which processes involving the reduction or elimination of chemical substances considered dangerous to human health and the environment are preferable, the biodegradable, biocompatible and non-toxic nature of hydrogels make these polymers a valuable alternative in the production of clean and renewable materials with unlimited applications, such as in the food industry, pharmaceuticals, tissue engineering and healthcare products [13]. Generally, hydrogel synthesis involves the use of inexpensive, readily available solvents and reagents in high purity from a variety of sources, and their combination with organic or inorganic compounds allows the creation of smart devices that can be used as catalysts for heavy metal removal from water, as capture agents for proteins and peptides, or even in biomarker analysis [14,15,16].

The synthesis of synthetic hydrogels allows control of the reaction parameters and te stoichiometry relative to the starting materials. Thus, it is possible to form well-defined structures with desirable characteristics for the intended application, such as long service life, high gel strength or high capacity of water absorption. Synthetic hydrogels are usually prepared using chemical polymerization techniques, in particular, thermal or photo-initiated polymerizations from monomers, including ethylene glycol, 2-hydroxyethyl methacrylate, vinyl acetate, *N*-*tert*-butylmethacrylamide (*t*BMAm) and acrylamide (AAm), among others. Natural hydrogels are formed using biodegradable polymers from natural sources such as proteins (collagen, fibrin or gelatin) or polysaccharides (alginate, chitosan or starch, for example) and exhibit considerable biodegradability and biocompatibility compared to synthetic hydrogels. The combination of natural and synthetic polymers gives rise to hybrid hydrogels: materials with unique characteristics capable of incorporating the advantages of both types of polymers, such as a good control of rigidity, biodegradability, high strength and viscosity [17]. 

**Figure 2:**
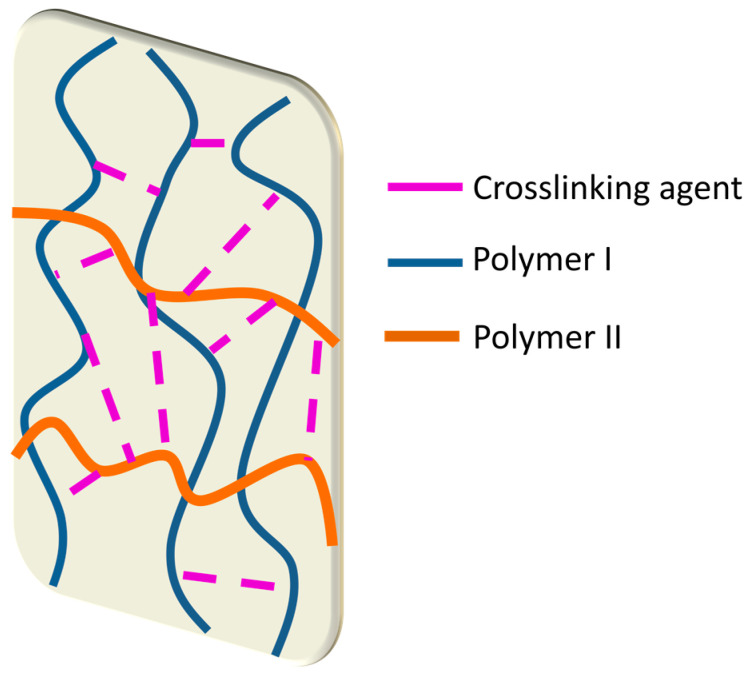
Structural model of an IPN hydrogel.

Homopolymeric hydrogels are formed using a particular class of monomers for the entire polymeric network and are capable of forming a crosslinked or non-crosslinked skeletal structure depending on the type of monomer and the polymerization method used. Mostly used as slow drug delivery devices and in contact lenses, non-crosslinked homopolymeric hydrogels are important in the biomedical field and in agriculture due to their high solubility in aqueous medium and adequate solubility in a variety of polar and non-polar solvents. The main representatives of this type of hydrogel are poly(ethylene glycol) (PEG), poly(*N*-vinyl-2-pyrrolidione) (PNVP), poly(vinyl alcohol) (PVA) and poly(acrylamide) (PAM). The combination of two or more monomeric species, with at least one being hydrophilic, gives rise to copolymeric hydrogels, which are partially soluble in aqueous medium and can show random structural configurations, block configurations or alternating configurations along the polymeric chain, in addition to being generally crosslinked by both covalent and ionic chemical bonds. Multi-polymer interpenetrating polymeric hydrogels, or IPN hydrogels (interpenetrating polymer network), are formed by combining at least two polymers by inserting a pre-polymerized hydrogel in a solution containing monomers and a reaction-initiating agent. The crosslinking process takes place independently for each polymeric component contained in the network. In this way, each polymer can maintain its responsiveness to external stimuli to form smart gels, capable of responding to two or more stimuli concurrently. IPNs can be formed by synthetic polymers as well as natural or hybrid polymers, with matrices much denser than conventional hydrogels, resulting in materials with improved mechanical properties [18]. Figure 2 shows one of the possible structural representations of IPN hydrogels formed by a crosslinking agent and two polymeric chains.

The morphological characteristics of hydrogels such as shape, size and the size distribution of pores are important parameters in the process of the diffusion and sedimentation of cells, proteins and nutrients inside and outside the gel. Non-crystalline, or amorphous hydrogels, are characterized by their lack of spatial ordering over long distances at the molecular level. These materials tend to exhibit gradual softening with increasing temperature and have great applicability in the pharmaceutical industry in healing procedures such as hydrogel wound dressing [19]. Usually, these gels are composed of water and glycerin-based products designed to assist in maintaining a moist environment in injured regions during the healing process. Amorphous hydrogels can promote granulation and epithelialization, in addition to facilitating autolytic debridement, a process in which the body uses its own enzymes and moisture under a dressing, causing the injured tissue to become liquefied and then absorbed by the gel itself [20]. Hydrogels with semi-crystalline structure are complex mixtures formed by crystalline and amorphous domains and present significant mechanical resistance and performance due to better energy dissipation in the gel network. In semicrystalline hydrogels, the crystalline phase consists of crosslink domains, which form ordered aggregates within the gel, allowing these materials the ability to abruptly change their mechanical properties reversibly when they reach the crystalline melting temperature (*T_m_*). Thus, contrary to what is observed with amorphous hydrogels, semicrystalline hydrogels quickly change their conformation from a solid-like to a liquid-like state, and can be applied in engineering and technology as smart inks for 3D or 4D printing, shape-memory hydrogels, in chemical motors or as injectable gels, among other applications [21]. Hydrogen-bonded hydrogels exhibit interactions that are stronger than dipole–dipole interactions obtained by lowering the pH in aqueous solutions containing polymers functionalized with carboxylic groups, such as carboxymethyl cellulose or polyacrylic acid. In both cases, the decrease in pH allows an increase in crosslinking in the polymeric network resulting from the formation of hydrogen bonds in the gel, forming low-energy structures due to the equilibrium between the enthalpy gain of hydrogen-bonding energy and the entropy loss of motion freedom. This technique is very common, for example, in the process of graft polymerization between poly(acrylic acid) and poly(ethylene oxide) (PAA-*g*-PEO) in aqueous media to form polymer chains with greater mobility that can be confined to a certain extent, and that result in the formation of supramolecular structures or aggregates [22]. Hydrocolloids are hydrophilic polymers of high molecular weight usually used as a functional ingredient in food formulations to provide a gain in texture, flavor, shelf life and the consistency of products, in addition to improving the gelling effect by controlling the product’s microstructure. With many hydroxyl groups in their structure, hydrocolloids can immobilize water molecules in their interior in a controlled manner to form a rigid structure that is resistant to the flow of nutrients, which gives these materials viscoelastic characteristics, behaving like a liquid and a solid simultaneously. The most widely used hydrocolloid gelling agents are pectin, gelatin, alginate, agar and gellan, while the most used hydrocolloid thickening agents are those derived from cellulose, starch, xanthan and some types of gum such as Arabic gum, guar gum, gum karaya, locust bean gum and gum tragacanth [23]. 

The type of crosslinking technique used for the synthesis of hydrogels determines the classification of these materials as physical or chemical hydrogels. Physical hydrogels are largely reversible and can be dissolved through changes in environmental conditions, such as temperature, ionic strength and pH. In the case of thermo-reversible physical gels, their reversibility process occurs without any occurrence of real hysteresis. Divided into two main categories, “strong” physical gels and “weak” physical gels, these polymers respond as solid-like at smaller deformations. “Strong” physical gels are characterized by remaining solid even at larger deformations, while “weak” physical gels are considered structured fluids which flow like liquids as a result of larger deformations. Governed by ionic interactions, hydrogen bonds or hydrophobic interactions, physical gels can be synthesized by applying crystallization methods or stereo-complex formation to form reticulated structures even at ambient conditions. One of the most important polymers in this category is the poloxamer (PX), a thermoreversible non-anionic block copolymer hydrogel usually synthesized in aqueous solution from hydrophobic poly (propylene oxide) (PPO) and hydrophilic poly (ethylene oxide) (PEO). In this reaction, spherical micelles are formed by increasing the temperature until the critical micelle temperature (CMT) is reached. If the temperature rises further, the micelles organize themselves through hydrophilic interactions to form gels. Other examples of physical hydrogels are those formed by polysaccharides such as dextran, chitosan and carboxy methyl curdlan physically reticulated by hydrophobic modification [24,25]. In chemical crosslinked hydrogels, also known as permanent hydrogels, the polymeric crosslinking takes place through covalent bonds joining different macromolecular multifunctional chains which result in highly crosslinked gels both in the dry state and in solution. Chemical crosslinked hydrogels are capable of covalently joining both natural and synthetic polymers through the interaction of functional groups dispersed in the polymeric structure, such as hydroxyl, carboxylic acid and amino groups, with multifunctional molecules such as acrylamide or dialdehyde (glutaraldehyde), for example, to form gels with a controlled time of freezing. The chemical synthesis of this class of polymers occurs through two main techniques: three-dimensional polymerization or the direct crosslinking of water-soluble polymers. In 3D polymerization, hydrophilic monomers are polymerized in the presence of multifunctional crosslinkers. However, this type of polymerization results in substantial amounts of unreacted monomers and byproducts which must be removed from the gel by an extensive purification process, taking days or weeks to complete. In consequence, to avoid the purification process, many researchers have used techniques of irradiation or heat treatment to serve as a subsequent post-polymerization curing, thus increasing the reaction yield. In this regard, the direct crosslinking of water-soluble polymers becomes a more interesting polymerization technique from the point of view of reaction economy, since aqueous soluble polymers are used directly without generating significant amounts of byproducts or unreacted monomers after synthesis, eliminating the need for purification [26].

The physical, chemical and mechanical properties of hydrogels can also be improved by the synthesis of multi-structured systems, such as in systems produced from the encapsulation of functional nanoparticles in the structure of smart nanogels. Examples of this are the works by Sabbagh et al., who synthesized κ-Carrageenan/NaCMC hydrogels by the sol–gel process containing Mg_0.99_Zn_0.01_O nanoparticles for the release of catechin, an important ingredient of green tea known for its antioxidant, antimicrobial, anticancer and antimutagenic properties. The authors observed that the presence of nanoparticles in the hydrogel structure increases its physical and mechanical strength, in addition to increasing the swelling ratio of hydrogels when mixed with carboxymethylcellulose (CMC) sodium salt and dispersed in distilled water. Furthermore, it was observed that the concentration of magnesium in the nanoparticles is directly related to the ionic transfer to the surface of the composite, since the ion works as a secondary electron donor, increasing the net charge on the surface of the particle, which allows an increase in the concentration of counter-ions in this region [27]. In another study, Sabbagh et al. added clays (montmorillonite, illite and kaolinite) to acrylamide hydrogels to form nanocomposites with greater water absorption capacity and greater surface area. The authors observed that the inclusion of clays in the hydrogel structure, in addition to affecting its biodegradability, allows a higher swelling rate in the composite, which may favor the release of drugs incorporated into the hydrogel network, in addition to favoring the release of agglomerated molecules more efficiently [28]. In addition to the biomedical field, these smart polymer composites have important ecological applications such as antifouling coverings on crystal substrates, water-harvesting materials for the removal of water droplets from moisture air, soluble polymer catalysts for the treatment of fissile waste and polluted biochemical devices, among others [29].

## 3. Bio-Monomers Used in the Synthesis of Hydrogels

Natural bio-based monomers derived from plants or animals are widely used in the synthesis of hydrogels and their compounds for the production of biopolymers and biomaterials that are biocompatible, biodegradable, non-toxic and of high porosity, characteristics much sought after in the biomedical field. These materials can undergo several chemical and physical modifications from the presence of an external stimulus, which makes them extremely versatile materials for the production of drug delivery systems, biochemical sensors, and as the main components in the production of biological scaffolds applicable to the area of tissue regeneration. Among the main natural bio-based monomers of this class are chitosan, glycolic acid, acrylic acid, lactic acid, ethylene glycol, propylene oxide, collagen, fibrin, fibrinogen, platelet-rich plasma, alginate, gelatin, albumin and hyaluronic acid, among others. Table 1 describes some of these natural bio-based monomers and biopolymers and their main applications.

### 3.1. Chitosan

**Figure 3:**
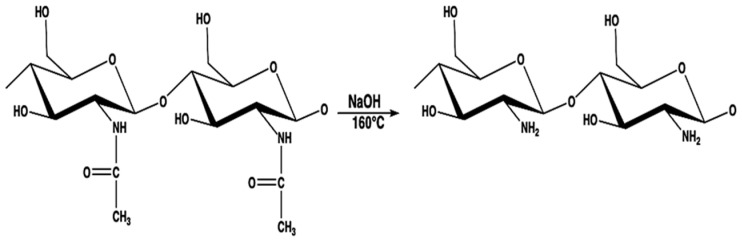
Deacetylation of chitin to obtain chitosan in alkaline medium.

Chitosan is an important polysaccharide produced through an alkaline deacetylation of chitin, which is found in crustacean exoskeletons, fungal cell walls and biological materials (Figure 3). Chitosan, by presenting free amino groups in its structure, is a molecule capable of forming stable complexes with metal cations, which makes it an important monomer for obtaining functional biopolymers with wide applicability in the biomedical industry [53]. Different methods of preparing chitosan result in different degrees of deacetylation, the distribution of acetyl groups, and changes in the viscosity and molecular weight of the polymer, in addition to promoting distinct degrees of polymerization. These are important parameters that influence not only the solubility of these biopolymers but also their antimicrobial activity, since in an acidic medium the amino groups present in the biopolymer are protonated, resulting in an overall positive charge (-NH_3_^+^). Thus, when in contact with the bacterial cell, chitosan is attracted by the negative charge of the bacterial cell wall, thus causing disruption of the cell and altering the membrane permeability. In addition, chitosan can bind to bacterial DNA, causing the inhibition of DNA replication and subsequently cell death. Obtaining chitosan from chitin depends on several factors, such as the reaction temperature, time, and concentration of sodium hydroxide. In common practice, at least 85% deacetylation is acquired for achieving a good solubility of chitosan [54].

### 3.2. Glycolic Acid (GA)

One of the constituents of sugarcane juice, glycolic acid, has great application in the materials and cosmetics industry, in addition to being an important indicator of hyperoxaluria syndromes characterized by a high predisposition to kidney stone formation. In patients diagnosed with type I hyperoxaluria, an increased urinary excretion of oxalic and glycolic acids is observed. In patients diagnosed with type II hyperoxaluria, there is an increase in oxalic acid and glyceric acid excretion. Thus, the concentration of glycolic acid in biological fluids is used as an index for the differential diagnosis of hyperoxaluria syndromes [55].

**Figure 4:**
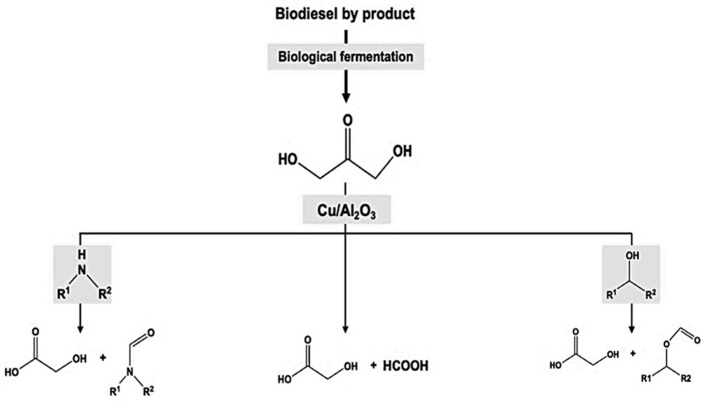
Illustrative diagram of one-pot synthesis method of producing GA, formamides and formates from 1,3-dihydroxyacetone catalyzed by Cu/Al_2_O_3_.

In a recent study, Dai et al. reported a one-pot synthesis method of producing GA from dihydroxyacetone (DHA) by using a non-noble metal-based catalyst [56]. This method also produced other versatile building blocks such as formamides and formates, as can be seen from Figure 4. In the presence of a clean oxidant, such as hydrogen peroxide, carbon–carbon bonds in DHA were selectively transformed to GA. The yields of GA were reported to be around 85% at room temperature.

Generally combined with lactic acid, glycolic acid is part of one of the most versatile copolymers in the biomedical industry: poly(lactic acid-*co*-glycolic acid) (PLGA). PLGA is a highly biodegradable and biocompatible aliphatic polyester used in drug delivery, tissue regeneration and growth, implants and fractures [57]. The polymerization process to obtain PLGA can take place from two polymeric routes: the polycondensation of lactic acid and glycolic acid to obtain a low-molecular-weight copolymer, or polymerization by opening cyclic dimers of lactic acid and glycolic acid, whose copolymers generally have a high molecular mass, resulting in better mechanical properties [58].

### 3.3. Acrylic Acid (AA)

Glycerol is an essential biomass-derived raw material and a significant byproduct of biodiesel production. In recent years, studies have tried to explore different conversion routes to synthesize various chemicals. Glycerol is abundantly available and compatible with principles of green chemistry because it is a biodegradable and sustainable raw material. One of the critical conversion processes is the dehydration–oxidation of glycerol to acrylic acid (AA) [59]. Acrylic acid polymers have a large number of hydrophilic groups in their structure, mainly hydroxyl groups and carboxylic acids. The hydrophilicity of these polymers makes them excellent candidates for hydrogel synthesis. The high water-retention capacity of acrylic acid-based polymers may be associated with the formation of hydrogen bonds involving the hydroxyl groups of the polymer main chain, which favors the formation of a continuous and strong network capable of retaining large amounts of water in its internal structure [60]. The synthetic route for obtaining AA usually occurs in a two-bed fixed-bed reactor system catalyzed by Cs_2.5_H_0.5_PW_12_O_40_ supported on Nb_2_O_5_ (CsPW-Nb). Furthermore, it is still possible to obtain AA via catalytic oxidation from vanadium–molybdenum oxides supported on vanadium−molybdenum mixed oxides supported on silicon carbide (VMo-SiC) [61].

**Figure 5:**
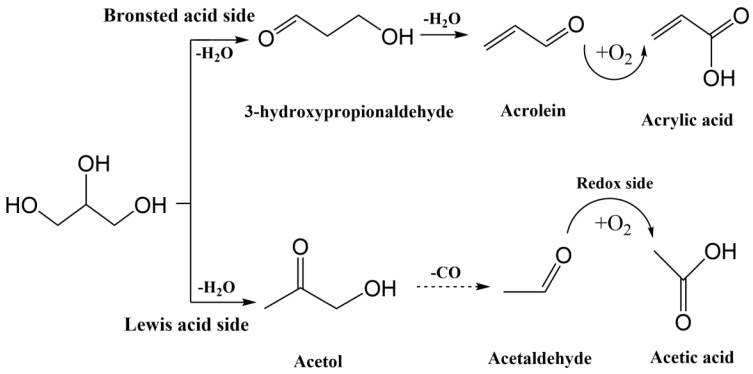
Proposed overall reaction pathways for glycerol dehydration representing the 1st and 2nd dehydration as well as tautomerization, according to the acrolein formation pathway.

A combination of a single bed with mixed catalysts and two beds loaded separately with the catalysts was also studied. It was observed that for single-bed configuration a yield of only 25% for AA was achieved, whereas a higher yield of 75% was obtained for AA in the two-bed system. Both catalysts, CsPW-Nb and VMo-SiC, had similar reaction conditions and oxygen ratios. An overall reaction pathway of the dehydration–oxidation of glycerol to acrylic acid is shown in Figure 5.

### 3.4. Lactic Acid (LA)

**Figure 6:**
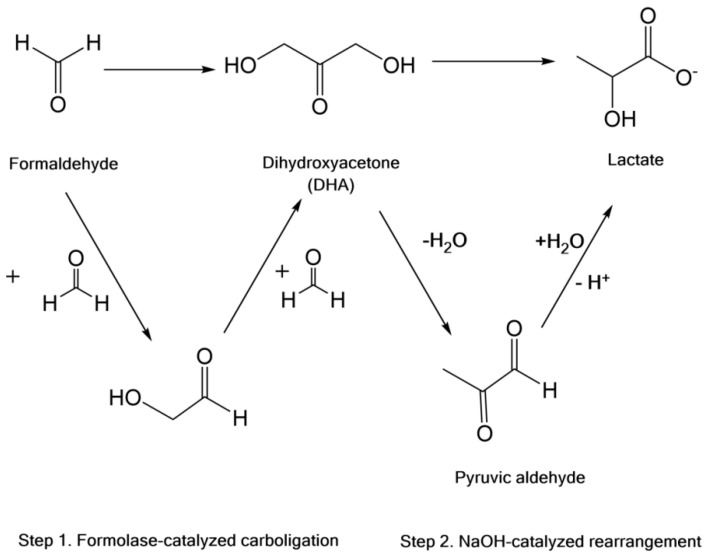
General two-step synthesis of lactate from formaldehyde.

Lactic acid is widely used in pharmaceuticals, cosmetics, food products, the dairy industry and polymer industries. It is generally used to produce many biodegradable polymers, such as polylactic acid, or as part of copolymers such as glycolic acid, ethylene glycol, ethylene and styrene, among others [62,63]. Lactic acid can be found in two optically active forms—*D*-lactic acid and *L*-lactic acid—as well as its enantiomeric form, *D*-*L*-lactic acid. The polymers from their polymerization have different physicochemical properties depending not only on the type of reaction applied to obtain them, but also on the relative percentage of their isomers in the polymer chain. When compared to glycolic acid polymers, lactic acid generally forms polymers with a low rate of degradation, good tensile strength and high mechanical strength, which makes it an excellent biomedical support [64]. The conventional chemical synthesis of lactic acid is carried out at high temperatures using costly metallic catalysis, such as catalysts based on tin, zinc, aluminum and lead [65]. It can also be produced by the microorganism-based fermentation of different sugars such as glucose, fructose, and cellulose. In recent years, the chemical synthesis of lactic acid from sugars and glycerol has attracted considerable attention. In this regard, Li et al. studied a green chemoenzymatic cascade synthesis scheme to transform C1 compounds (e.g., formaldehyde) into lactic acid [66]. In this method, a newly identified variant of formolase is used in the presence of sodium hydroxide as a catalyst. An overall yield of around 83% was obtained with 100% atom economy in ambient conditions, and the formation of dihydroxyacetone (DHA) as an intermediate product. The reaction scheme can be seen in Figure 6.

### 3.5. Ethylene Glycol (EG)

**Figure 7:**
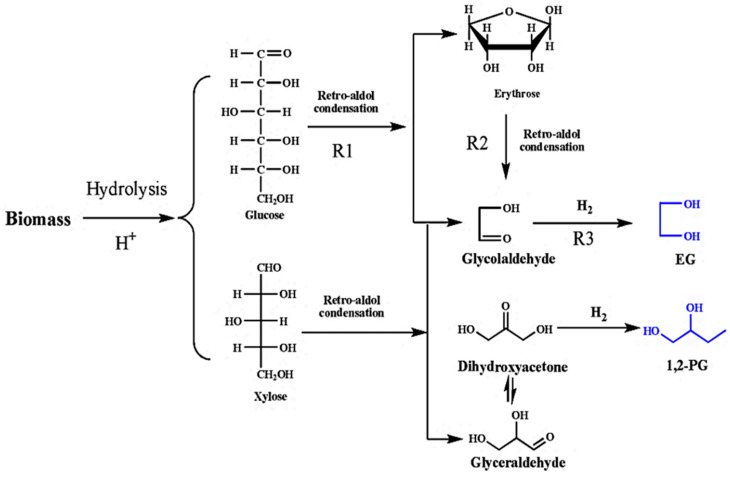
Illustrative diagram of the reaction for conversion of cellulose and hemicellulose to EG.

Ethylene glycol (EG) is generally obtained from the reaction of ethylene oxide with water under basic or acid catalysis at neutral pH under elevated temperatures. However, EG can also be produced from biomass through several routes. Important routes include converting bio-oil, sugarcane, or corn stover to EG through multiple steps. Another method is the direct conversion of cellulosic biomass to glucose, which is then subsequently converted to EG. Common lignocellulosic biomass such as corn stalk, popular wood, and miscanthus are commonly used. In this route, several catalysts have been used, such as Ni-W/SBA-15, which gives a high yield of around 76%. Similarly, superior performance was exhibited by the Raney Ni–tungstic acid catalyst, which possesses great potential for the commercial-scale conversion of cellulosic biomass [67]. The conversion scheme of biomass to EG is shown in Figure 7.

### 3.6. Propylene Oxide (PO)

**Figure 8:**
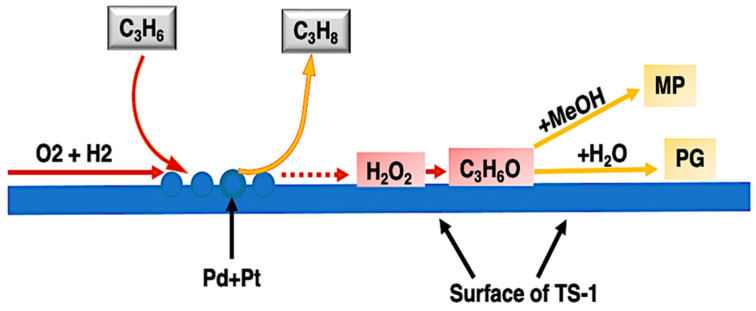
Illustrative scheme of a one-pot green route to produce propylene oxide catalyzed by 0.2% Pd-0.02% Pt catalyst supported over TS-1 zeolite using liquid or supercritical carbon dioxide as a solvent.

Propylene oxide (PO) is considered one of the most important chemical compounds in the world because it is an intermediate compound used to produce different products such as polyether polyols for the polyurethane industry, cosmetics, agricultural products, lubricants, antifoam agents, adhesives and inks, among others [68]. Usually combined with ethylene oxide, propylene oxide produces hydrogels with hydrophobic groups on its surface and is extremely useful in the area of bioengineering, since its high protein resistance reduces the non-specific adsorption of proteins on different surfaces [69]. PO can be produced from two main routes: the oxidation of isobutane or ethylbenzene to the corresponding hydroperoxide, which involves the usage of ethylbenzene hydroperoxides or *tert*-butyl to oxidize propylene, or from the chlorohydrin process, where propylene is combined with chlorine in the presence of water and a base, generating PO and large quantities of salts as byproducts. Although these synthetic routes are the most applied around the world for obtaining PO, they mostly occur in the presence of toxic organic solvents with low recoverability. Chen and Beckman developed a one-pot green route to epoxidize propylene producing PO using in situ-generated hydrogen peroxide [70]. The process was carried out in the presence of a 0.2% Pd.02% Pt catalyst supported over TS-1 zeolite using liquid or supercritical carbon dioxide as a solvent. A yield of 23% for PO with a selectivity of 82% was obtained at a temperature of 60 °C using ammonium acetate as an inhibitor which resulted in an effective suppression in the production of reaction byproducts. The conversion scheme observed by Chen and Beckman is illustrated in Figure 8.

## 4. Hydrogel Nanoparticles

**Figure 9:**
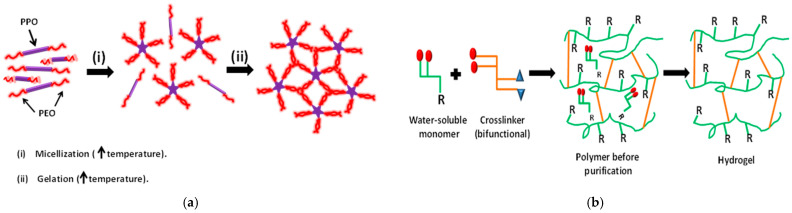
Illustrative representation of (**a**) physical gelation process of poloxamer and (**b**) chemical hydrogels synthesized by 3D polymerization.

According to the IUPAC (International Union of Pure and Applied Chemistry) nanogels are defined as a particle of gel of any shape with a size ranging between 1 and 100 nm in diameter, while microgels are those whose size varies between 100 nm to 100 μm in diameter, approximately. However, many authors use nomenclature nanogels, polymeric nanogels, microgels or crosslinked micelles to refer to hydrogel nanoparticles with sizes varying between 1 and 100 μm in diameter, since the synthesis methods of these polymers are practically the same [71]. The miniaturization of hydrogels for the nano- or micrometric range has expanded its field of application to different areas of science such as drug delivery systems, cell-based therapies, nanoreactors, biomimetic mechanical devices, tissue engineering or liquid microlenses, among others. These materials have unique properties due to their ability to combine the advantages of hydrogels in the same system, such as high porosity, soft consistency, swelling properties, hydrophilicity, absorbability, biocompatibility, flexibility, viscosity and controlled degradability, with properties of nanoparticulate systems such as very small size, increased stability of therapeutic agents against degradation, functionality dependent on shape and size, time-controlled delivery and site-specific activity [72]. In addition, nanogels can associate through chemical or physical interactions with small molecules such as drugs, proteins, peptides, oligosaccharides, nucleic acids, and antigens, among others. Presenting considerable stability, biologic consistence, drug loading ability, good permeability to different bioactive agents and ability to respond to external stimuli such as temperature, pH, light and electricity, nanogels are part of the group known as smart materials with a wide spectrum of application [73]. Examples of the physical gelation process and chemical 3D polymerization can be seen in Figure 9.

Another important feature of nanogels is that, in addition to swelling and shrinking much faster than macroscopic gels, they can be incorporated into organic or inorganic materials such as silver nanoparticles, inorganic molecules, magnetic nanoparticles, carbon nanotubes, alginate or gold nanoparticles, for example, to form nanocomposites with unique properties. An example of this is the work of Zhang et al. which incorporated PEGylated gold nanorods (GNRs) and paclitaxel in temperature-responsive chitosan polymeric micelles (PTX-M) to form a nanocomposite with photothermal and chemotherapeutic properties; exposure to laser irradiation induced photothermal damage mediated by GNRs previously confined in tumor cells, releasing the drug directly into the target tissue and allowing a more effective chemotherapy treatment with greater bioavailability [74]. In the same vein, some authors have used alternating magnetic fields (MFAs) to increase the temperature in a specific way in nanocomposites containing magnetic nanoparticles in order to release bioactive compounds dispersed in the polymeric matrix through Neel and Brownian relaxations, for example [75]. In drug delivery systems, nanogels are capable of carrying a wide variety of drugs formed by hydrophilic or hydrophobic molecules with different molecular weights, presenting as the main advantages: high drug loading capacity; longer circulation time in the bloodstream without being captured by macrophages; better permeation via biological membranes, tissues and capillaries; and the ability to be easily recognized by cells in addition to controllable biodegradability and the release of nontoxic metabolites [76]. 

Nanogels are classified according to their structural properties, which are governed based on the method of crosslinking used during synthesis and the nature of their physical or chemical interactions (covalent bonds). Thus, the synthetic methods used for the formation of polymeric gels, especially at the nanoscale, must consider all techniques that result in controlling the size and shape of the synthesized particles. The chemical and physical methods previously mentioned to produce hydrogels are also valid for the synthesis of nanogels, i.e., chemical methods form stable or rigid structures while physical methods form more flexible and collapsible structures as a result of changes in their environment. Among the best known approaches for the synthesis of nanometric materials are the “top–down” approach, in which mechanical methods are used to miniaturize the raw material to nano-size (lithography, for example) and whose application is more suitable for synthesizing micron-sized particles, and the “bottom–up” or self-assembly approach, in which atoms and molecules are manipulated in order to create nanostructures through non-covalent interactions such as electrostatic and/or hydrophobic associations to form structures such as nanowires, nanotubes, nanodots, self-assembly and positional assembly, for example. If considered individually, interactions in the “bottom–up” approach are weak, but the continuous process of self-assembly forms structures with many interactions involved, resulting in nanoparticles that are stable and easily manipulated. With respect to nanogels, the “bottom–up” approach is the most used method due to the ease of preparation, the high efficiency in the incorporation of bioactive agents and the possibility of chemical modification with different types of molecules and functional groups, improving nanogel stability and targetability by the formation of more homogeneous and controllable size particles [73]. 

The most-used reaction methods for the manufacture of nanogels can be grouped into five major categories: preparation from polymer precursors; preparation via monomer polymerization; the crosslinking of preformed polymers; the photochemical approach; and pullulan chemistry modification. In the preparation of nanogels from polymer precursors, amphiphilic copolymers are structured by self-assembly through functional groups arranged along the macromolecular chain. By blocking this assembly, polymeric precursors derived from polymerizable groups can be covalently crosslinked, resulting in crosslinked nanogels with size adjustable by varying the concentration of the polymer, and by its thermoresponsive behavior based on its lower critical solution temperature (LCST). The presence of functional groups such as disulfide, amide or imine in polymeric precursors and the possibility of applying click chemistry or photo-induced crosslinking reactions are some of the strategies for the synthesis of nanogels starting from polymer precursors [48]. Table 2 lists the main synthesis techniques to produce nanogels from polymer precursors, the main characteristics of the particles and some of their most common applications.

The preparation of nanogels via monomer polymerization is one of the most common types of synthesis and occurs through the direct polymerization of a homogeneous or heterogeneous mixture of different types of monomers that contain at least one polymerizable functional group. This polymerization, usually originated through radical processes, can be achieved both by dissociation of the monomer and by the degradation of chemical initiators upon the absorption of UV light by a photoinitiator, or even by the radiolysis of water via ionizing radiation [89,90,91]. One of the most important techniques associated with the preparation of polymers via monomer polymerization is the technique known as reversible addition−fragmentation chain-transfer, or RAFT polymerization. This technique, first reported in 1998 by Rizzardo et al., is based on the balance between active and dormant chains whose mechanisms begin with the formation of radical monomeric species which are added to the RAFT agent (chain-transfer agent (CTA)). During the propagation stage, the CTA’s functional chain end-group, typically the thiocarbonylthio group, is reversibly transferred between dormant chains (macroRAFT agent or macroCTA) and propagating radicals. In this process, the speed of the equilibrium rate between the addition and fragmentation processes is greater than the speed of the propagation step, causing a very low number of monomer units to be added per activation cycle, which implies a highly controlled polymerization process with a similar degree of polymerization (DP) at a given time [92,93]. Kumar and Binder used RAFT polymerization to couple the central peptide sequence of the amyloid-β (Aβ) protein Leu-Val-Phe-Phe (LVFF, Aβ17–20) to homopolymers, providing LVFF–polymer conjugates and the formation of LVFF-functional polymeric hydrogels [94]. According to the authors, the amyloidogenic peptide Aβ17–20 is identified as the key sequence critical for the fibrillation of the native Aβ1–40/42 protein, which is directly associated with neurodegenerative diseases such as Alzheimer’s disease (AD), for example. The authors observed that the RAFT polymerization technique was essential to provide well-defined homopolymer/peptide conjugates with a high swelling degree at equilibrium for the peptidic hydrogel in aqueous medium (greater than 450%). In addition, the authors describe that the functionalized hydrogel obtained tends to form micellar aggregates with an average diameter of 25 nm, probably due to the presence of hydrophobic amyloidogenic peptides and hydrophilic polymeric segments, which can behave as mimetics of functionalized amphiphilic block copolymers. This is highly interesting for micellar drug delivery applications or 3D cellular encapsulation.

The use of ionizing radiation in polymerization processes, a technique known as photopolymerization or photochemical polymerization, has become an extremely important technique in organic chemistry due to its ability to activate micro- or macromolecules without the need to use extra reagents that could result in the formation of byproducts capable of affecting the final quality of the polymer [95]. Furthermore, the use of gamma rays or a high-energy electron beam in chemical processes tends to allow obtaining compounds with remarkable stereo- and regioselectivity without the need to apply high temperatures or pressure during the reaction process [96].

**Figure 10:**
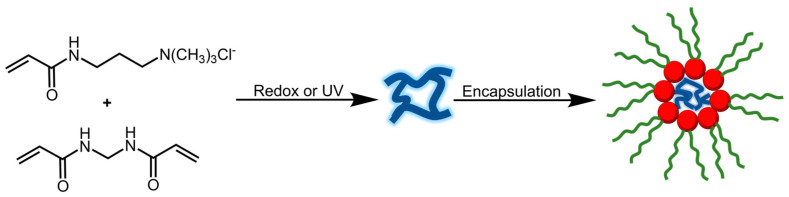
Representation of a photochemical reaction model for obtaining nanogels catalyzed by photoinitiators in the presence of crosslinking agents.

The formation of active sites in photochemical polymerization processes in aqueous media begins with the formation of reactive radical species in the polymeric chain. The combination of macroradicals (formation of hydroxyl radicals during the process of radiolysis of water molecules and their attack on polymeric chains) at different points in the polymer allows the formation of crosslinked structures resulting from covalent bonds, obtaining hydrogels that are relatively pure and, in some cases, of high structural complexity [97]. An example of this is the photopolymerization reaction of (3-Acrylamidopropyl)-trimethylammonium chloride (APTMACl) using *N*,*N*′-methylenebisacrylamide as a crosslinking agent in the presence of surfactants to obtain cationic crosslinked polymers which can be encapsulated by inverse micelles for the formation of nanogels (Figure 10).

**Figure 11:**
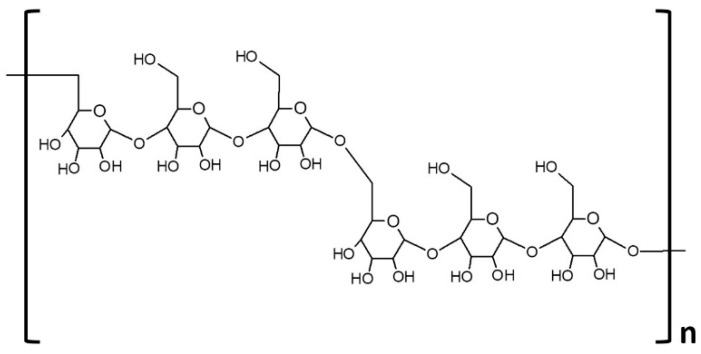
Representation of the pullulan chemical structure.

Recently, the pullulan polysaccharide has gained great attention as a functional material in both the biotechnological and biomedical fields due to its ease in incorporating hydrophobic chains, such as cholesterol, resulting in an amphiphilic polymeric material which can act as an excellent nanocell carrier of high biocompatibility and biodegradability and low toxicity [98]. Pullulans are generally synthesized by the yeast-like fungus *Aureobasidium pullulans* with long carbon chains from the polymerization of hundreds of repeated units of maltotriose trimer *α*-d-glucopyranosyl-(1,6)-*α*-d-glucopyranosyl-(1,4)-*α*-d-glucopyranosyl-(1,4), as shown in Figure 11. Despite presenting different active sites available for functionalization and graphitization, some authors suggest that the hydroxyl groups at the C-6 position in the carbon chain of the pullulan molecule appear to be more accessible, which would justify the great preference for these groups during chemical modification processes [99]. Pullulan-based hybrid hydrogels tend to present smart stimuli responses in ambient conditions, which enables their use in different areas of biomedicine such as tissue engineering, cancer therapy, protein delivery, imaging and vaccine development, for example [100].

**Scheme 1:**
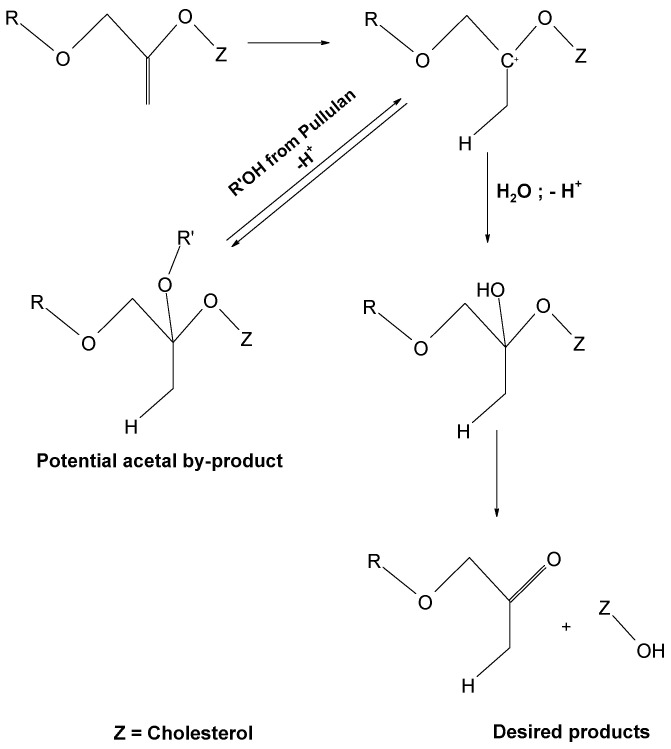
Proposed scheme to explain the kinetics of cholesterol/pullulan polymerization under acidic conditions from intramolecular trapping of the protonated cholesteryl vinyl ether.

Morimoto et al. prepared a self-assembled nanogel from pullulan modified with acid-labile cholesterol (acL-CHP) at neutral pH and tested its behavior as a protein delivery vehicle under acid conditions [101]. Using protein cargo fluorescently labeled albumin (FITC-BSA) as a model, the authors observed that in 2 h of contact between the acL-CHP nanogel and FITC-BSA at pH 7.4, approximately 80% of the protein was incorporated for the formation of the acL-CHP/ FITC-BSA nanogel complex. When the pH of the medium was reduced from pH 7.4 to pH 4.0 (25 °C), results from the refractive index detector (RI or RID) chromatography revealed that approximately 27% of the FITC-BSA was released from the nanogel within 26 h via the acid-catalyzed hydrolysis of the nanogel. The authors suggest that the slow release of the protein under acid catalysis conditions at room temperature is probably the result of the formation of acetal byproducts from the intramolecular trapping of the protonated cholesteryl vinyl ether during hydrolysis, which can generate more stable protein/nanogel complexes, thus releasing the protein under acidic conditions (Scheme 1).

Polyesters derived from lactic acid, glycolic acid and caprolactone have great commercial and biomedical importance due to their controlled rate of degradation and the possibility of obtaining them from natural sources such as through bacterial fermentation, oxidative processes using microorganisms, or even from renewable catalytic processes, such as the use of zeolites, silica gel or alumina, in addition to natural clay as acid catalysts in several organic reactions [102,103,104,105].

**Figure 12:**
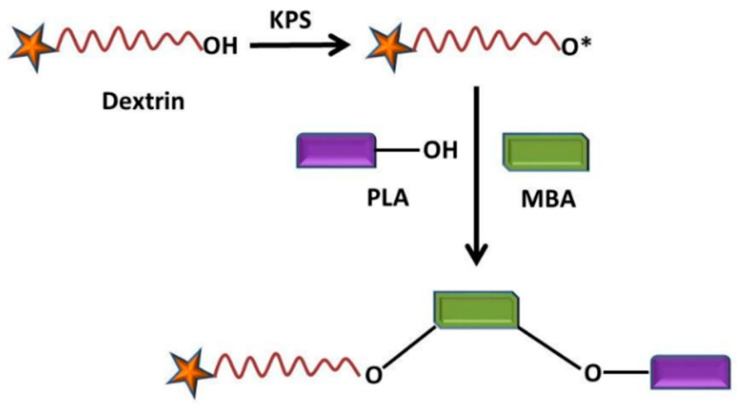
Process for obtaining PLA-based hydrogel by free radical polymerization using MBA as a crosslinking agent.

Widely used by the food and cosmetics industry, lactic acid and its polymerization products have gained prominence in the biomedical industry. Due to its controlled biodegradability and high biocompatibility, poly(lactic acid) (PLA) has become one of the most important polymers in the academic field, having mechanical and biological properties in addition to thermoplastic processability, and is studied in detail in order to obtain more versatile polymers that are easily processed and modified [106]. The preparation of PLA-based hydrogels considers not only the types of crosslinking agents used but also the main intermolecular forces present between the PLA chains (hydrogen bonding and hydrophobic/ionic interactions). One of the most common methods of obtaining PLA-based hydrogels is through crosslinking via free radical polymerization. These reactions are characterized by the formation of radical species from the addition of an initiator, usually potassium persulfate (KPS), whose propagation step results in the formation of long polymeric chains until the termination step is completed. Das et al. applied the free radical polymerization technique for the synthesis of hydrophobic polylactic acid (PLA) and hydrophilic dextrin hydrogel in the presence of *N*,*N*-methylene bisacrylamide (MBA) as a crosslinking agent and studied its effect on the controlled release of model drugs such as ciprofloxacin and ornidazole, as shown illustratively in Figure 12 [107]. The authors observed that the drug release profile follows first-order kinetics with higher R2 value and a non-Fickian diffusion mechanism, with the hydrophilic/hydrophobic segments as well as the crosslinker moiety in the network structure controlling the swelling ratio, thus allowing better control in drug release rates at temperatures close to 37 °C at pH 7.4.

## 5. Hydrogel–Biomolecule Interactions

Ultimately, the use of hydrogels for biochemistry applications relies on the control of hydrogel–biomolecule interactions. Interactions may be classified as electrostatic, hydrogen bonding, van der Waals, or hydrophobic. Salt bridges involving both hydrogen bonding and electrostatic interactions may also be possible. Hydrogen bonding may be less important than van der Waals interactions, since proteins can already satisfy hydrogen bonding requirements with water, but hydrogen bonding can enhance interaction specificity [108]. Hydrophobic interactions may be thought of as an entropic effect and may promote protein unfolding [109]. For a given system, variables governing which interactions are possible or dominant include the structure and surface charge of both the hydrogel and biomolecule, as well as temperature, pH, and composition of the surrounding matrix [110]. In terms of hydrogel–biomolecule interactions, the role of many interacting factors makes the characterization and control of protein–particle interactions a challenge, though surface chemistry complementarity in terms of hydrophobicity and surface charge distribution appears to emerge as a key consideration [108]. However, hydrogel surface chemistry and charge are, in turn, affected by protein adsorption [111]. Furthermore, even for hydrogels with similar charge characteristics, the nature of the binding interaction with biomolecules may vary, for example, as to whether entropic or enthalpic effects dominate. In general, increased particle hydrophobicity is associated with stronger interactions with biomolecules [112].

From the perspective of hydrogel formulation, biomolecule selectivity may be tuned by adjusting monomer identity and relative proportions. For example, NIPAM-based particles with 20% acrylic acid (AAc), 40% *N*-*tert-*butylacrylamide (*t*BA), and 2% *N*,*N*′-methylenebisacrylamide (BIS) were developed to bind the Fc domain of IgG, whereas particles with identical acrylic acid content in the monomer feed but incorporating 20–40% aromatic monomers were employed to neutralize peptide toxins [112]. Honda et al. demonstrated that properties of the final hydrogel depend not only on the identity and proportion of charged monomers but also on polymerization pH. The basis for their study was the idea that monomers that are not ionized at the polymerization pH would be imprinted in hydrophobic regions. Such monomers would be expected to exhibit less tendency to dissociate in the final hydrogel, affecting electrostatically driven protein interactions. NIPAM-based nanoparticles incorporating AAc as an acidic group or *N*-3-[(dimethylamino)propyl]-methacrylamide (DMAPM) as a basic group exhibited a substantially reduced binding of proteins with complementary charge when polymerized at a pH below or above monomer pKa, respectively [113]. However, the same hydrogel may exhibit different surface characteristics depending on environmental factors. The polymer lower critical solution temperature (LCST) may be thought of as the “hydrophilic–hydrophobic transition” from the polymer being soluble in water to preferentially forming polymer–polymer instead of polymer–water interactions. The pH of the surrounding medium is also relevant in the case of polymers with weak polyelectrolyte or polyampholyte groups. Within the pH range at which the groups are charged, the polymer exhibits a swollen state but transitions to a hydrophobic or collapsed state at pH values where the groups are neutral [114].

The control of electrostatically driven interactions with proteins involves the incorporation of ionizable monomers, since it considers the pH and ionic strength of the environment in which the hydrogel will be used. Nagy-Smith et al. used peptide building blocks with a charge of either +5 or -5 to develop self-assembling hydrogels [115]. The high hydrogel charge was designed to mitigate the weakening of electrostatic interactions under high salt physiological conditions, retaining control over protein release based on electrostatic interactions. Basu et al. observed that electrostatic interactions were important for the protein loading of anionic Ca^2+^-crosslinked wood-based nanofibrillated cellulose hydrogels [116]. The loading of positively charged lysozyme exceeded that of negatively charged bovine serum albumin (BSA) and fibrinogen. Protein charge also affected diffusion characteristics, since lysozyme, though small, exhibited a slow rate of diffusion comparable to large proteins with negative charge. In a computational study of protein and pH-responsive hydrogel interactions, Longo et al. proposed that the deprotonation of hydrogel carboxylic acid groups leading to local pH decreases could be applied to separate binary or ternary protein mixtures based on differences in amino acid protonation. More highly protonated proteins would be expected to exhibit increased adsorption to the hydrogel [117].

Although adsorption is controlled by long-range electrostatic interactions, protein desorption from hydrogels depends on short-range van der Waals and hydrophobic interactions, as well as hydrogen bonding. Therefore, the hydrogel structural changes that result from functional group ionization, rather than changes in the strength of electrostatic interactions, may be more important in dictating the protein–hydrogel interaction in certain cases. Dutta et al. studied lysozyme interaction with a NIPAM-*co*-allylamine hydrogel using single-particle tracking and fluorescence correlation spectroscopy super-resolution optical fluctuation imaging (SOFI) [118]. Results indicated that lysozyme desorption was controlled by heterogeneity in hydrogel size and structure, especially at pH 7.3 when the hydrogel was swollen and both the protein and hydrogel were protonated. Vagias et al. also observed the importance of hydrogel size and structure in governing IgG transport within an NIPAM-*co*-methacrylic acid hydrogel, concluding that repulsive electrostatic interactions dominated when both species were negatively charged [119]. However, though electrostatic, hydrophobic, and excluded volume effects determined the partitioning of the protein between bulk solution and hydrogel, the authors suggest that the rate of diffusion of the protein in the hydrogel was dictated by the hydrogel volume fraction.

Similarly, Zhou et al. observed that hydrogel mesh size was a key factor governing the diffusion behavior of lysozyme, IgG and bovine serum albumin (BSA) in zwitterionic poly(sulfobetaine methacrylate) (*p*SBMA) hydrogels [120]. Mesh size was increased by decreasing crosslink density and increasing ionic strength, since the latter effect disrupted intramolecular associations between zwitterionic groups in the hydrogel. At higher mesh size, diffusion behavior differed among the three proteins, with higher diffusion coefficients observed for smaller proteins. The authors indicated that similar diffusion coefficients were observed for all proteins at the highest crosslink density, possibly signaling very slow or no diffusion as a result of the small mesh size. Wu et al. also examined diffusion behavior in *p*SBMA hydrogels, comparing interactions of lysozyme and BSA in these polyzwitterionic hydrogels with PEG-based hydrogels [121]. The link between hydrogel structure and diffusion was also apparent in that proteins exhibited slower diffusion in hydrogels with greater PEG content and decreased free water space.

From the perspective of the biomolecule, Adroher-Benitez et al. developed a computational model to evaluate the interaction of a single biomolecule with a hydrogel network [122]. The results indicated that biomolecule hydrophobicity in addition to dipole moment are key variables governing the strength and nature of the interaction. The dipolar electrostatic attraction between the biomolecule and hydrogel increased with increased biomolecule dipole moment. Moreover, the increased hydrophobicity of the biomolecule seemed to increase the favorability of the interaction with the polymer. Accordingly, combining computational models with experimental procedures, it is possible to conclude that biomolecule–polymer interactions cannot be predicted based on only one single factor.

Protein structure may also be stabilized or denatured upon interaction with the hydrogel, depending on hydrogel surface chemistry and structure. Kisley et al. applied fast relaxation imaging to study the distribution and stability of phosphoglycerate kinase in polyacrylamide hydrogels on a localized scale. The results indicated that protein–hydrogel surface interactions, rather than the degree of protein confinement, were most relevant for protein behavior within the gel, since the stabilization of protein structure was observed at both 4 and 10% crosslinking [123]. Kabir et al. studied the enzyme refolding efficiency of hydrogels synthesized from vitamin B5 analogous methacrylamide (B5AMA), which function as thermoresponsive synthetic chaperones [124]. The results suggested that the hydrogen bonding interactions between the polymer and residual water were important for hydrogel thermal flexibility, which promotes enzyme refolding. The incorporation of ionic or hydrophobic monomers reduced the restoration of enzyme activity, possibly by constraining enzyme structural flexibility. The enzyme–polymer interaction appeared to involve the polymer surface rather than enzyme confinement to pores. Sen-Britain et al. focused on surface interactions to evaluate the effect of (hydroxyethyl)methacrylate (HEMA) hydrogels on the structural stability of the keratinocyte growth factor (KGF) [125]. Structural stabilization was observed for more hydrophobic surfaces incorporating methyl methacrylate, whereas unfolding occurred upon interaction with hydrophilic HEMA-*co*-methacrylic acid surfaces, likely due to hydrogen bonding.

**Figure 13:**
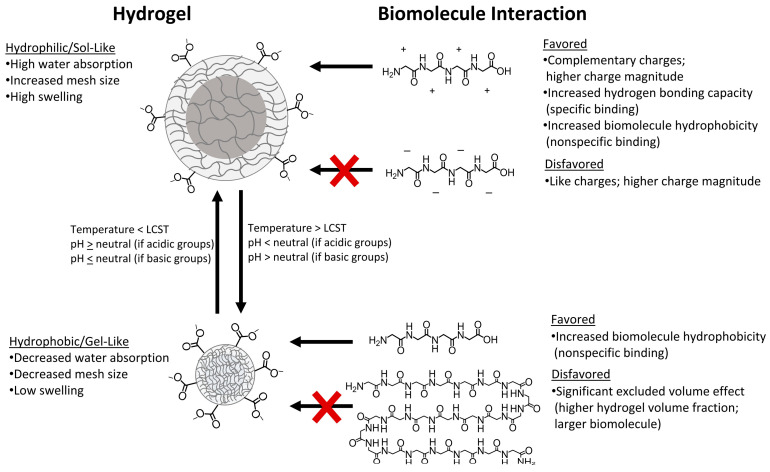
Hydrogel physicochemical properties and biomolecule interaction. General considerations are summarized, though the strength and nature of an interaction depend on several interrelated factors and vary by system.

In addition to affecting protein structure, increased hydrogen bonding capacity may strengthen hydrogel–protein interactions. Shin et al. observed superior protein binding to sodium hyaluronate hydrogels enriched in gallol groups compared to alginate beads. The increased protein binding was attributed to the intermolecular hydrogen bonding capacity of gallol groups [126]. Lanzalaco et al. noted that the incorporation of polysaccharides in hydrogels strengthened hydrogel–drug interactions via increased hydrogen bonding, though this may result in an undesired inhibition of drug release [127]. As noted by O’Brien et al., increased hydrogel hydrophobicity generally strengthens interactions with biomolecules, whereas, as noted by Yang et al., hydrogen bonding may be most important for enhancing interaction specificity [108,111,112]. Recent studies are consistent with these general trends if increased hydrogel and biomolecule hydrophobicity are interpreted as strengthening nonspecific binding, whereas increased hydrogen bonding is interpreted as strengthening specific interactions. Results also underscore the need to balance multiple factors, both general and system-specific, to engineer targeted hydrogel–biomolecule interactions. A schematic representation of the physicochemical properties of hydrogels and their different types of interactions with biomolecules can be seen in Figure 13.

## 6. Synthesis Strategies to Achieve Target Properties

The main strategy for hydrogel synthesis is aqueous precipitation polymerization. In addition to free radical polymerization, reversible addition–fragmentation chain-transfer (RAFT) and atom transfer radical polymerization (ATRP) have been used to achieve greater structural control and uniformity [128]. However, heterogeneous emulsion polymerization has been employed to control particle size within the micro- and nanoscale range [129,130]. To control hydrogel porosity, mesh size, and degree of swelling, degree of crosslinking is adjusted by changing the proportion of chemical crosslinker in the monomer feed or the relative proportions of physically crosslinked copolymers. In general, lower crosslinking density biases the hydrogel to the sol state—greater hydrophilicity, degree of swelling, and larger pore sizes—whereas higher crosslinking density biases the hydrogel to the gel state—greater hydrophobicity, more structurally collapsed, and greater mechanical strength [120,126,131,132,133]. When an especially high proportion of chemical crosslinker is used, structural heterogeneity may be introduced, since crosslinkers may be more rapidly incorporated in the growing polymer than other monomers, though this has not always been observed [134]. Even in the case of RAFT polymerization, evidence of spatial defects in the polymer structure has been observed [130]. For higher resolution structural control, the use of a mask with photolithography and stop-flow lithography permits the synthesis of hydrogels with localized, controlled geometry [134,135,136]. Moreover, the molecular imprinting technique has been used to synthesize hydrogels with structural complementarity to biomolecules by including the target biomolecule in the reaction solution during polymerization [137,138].

The ability of hydrogels to change structure in response to external stimuli is valuable for applications that rely on controlled biomolecule binding and release. Stimuli-responsive structural flexibility has been achieved by incorporating NIPAM in the monomer feed to yield thermoresponsive hydrogels [119,139,140]. Though the LCST of *p*NIPAM homopolymer is between room and body temperature, when additional monomers are introduced, the LCST may shift, with increased hydrophobicity often corresponding to a decrease in LCST. As noted above, hydrogels have also been developed to function as synthetic chaperones that guide protein refolding via heat-induced structural changes. Hydrogels for which structural changes are linked to pH have been synthesized by incorporating functional monomers with carboxylic acid, sulfonic acid, or amine groups. Specific examples of acidic monomers include acrylic acid, methacrylic acid, and 2-acrylamido-2-methyl-1-propanesulfonic acid, whereas basic monomers that have been employed include allylamine, [2-(methacryloyloxy)ethyl]trimethylammonium chloride, and *N*-3-[(dimethylamino)propyl]methacrylamide [141,142,143,144]. By incorporating zwitterionic monomers, in the case of poly(sulfobetaine) hydrogels, swelling is induced at increased ionic strength [103]. More targeted stimuli-induced structural changes have been achieved by incorporating enzyme substrates, and even enzyme–substrate complexes, within hydrogels. These systems have been engineered such that interaction with enzymes, competing substrates, or even a specific non-enzymatic protein triggers the cleavage of covalent bonds or the disruption of physical crosslinks in the hydrogel [145,146,147].

The optimal structural flexibility depends on the intended application of the hydrogel. In cases where enhanced mechanical strength is needed, strategies such as the design of dual crosslink networks or the incorporation of other polymer components such as polysaccharides have been used [144,146,148]. However, for a given formulation, mechanical properties may be controlled by increasing the mol% crosslinker or increasing photoactivation time in the case of photoinitiated polymerization, or increasing buffer pH in the case of proton transfer polymerization [149,150]. Another strategy is to use the hydrogel as a coating, combining the advantages of the biomolecule binding capacity and stimuli responsiveness of the hydrogel with the mechanical stability of a rigid substrate [151,152]. Hydrogels capable of self-healing after structural degradation have been engineered by the incorporation of disulfide links [152].

In addition to structural considerations, the degree of hydrophilicity or hydrophobicity of the hydrogel surface affects biomolecule interactions. Hydrophilicity may be increased by increasing the proportion of charged or polar monomers, whereas increasing the mol% of hydrophobic crosslinkers such as *N*,*N*′-methylenebisacrylamide or monomers such as *N*-*tert*-butylacrylamide has the opposite effect [153]. The incorporation of hydrophilic PEG and zwitterionic sulfobetaine components may result in the formation of a hydration layer that protects the hydrogel from biofouling. Furthermore, cationic groups such as quaternary ammonium, sulfonium groups, or protonated amino groups of chitosan may impart antibacterial properties to the hydrogel [152,153].

### 6.1. Synthesis with Water as a Solvent

As summarized above, a variety of techniques employing a range of starting materials, crosslinking strategies, and solvents have been successfully implemented for hydrogel synthesis for biomedical applications. However, the options for water-based synthesis make hydrogel materials particularly suitable for synthesis in accordance with the principles of green chemistry. Hwang et al. demonstrated the utility of thiol-epoxy click chemistry methods for hydrogel synthesis, focusing on base-catalyzed or photoinitiated polymerization from water or aqueous buffer [149]. Applied in various fields of science, including drug discovery, polymers, materials science, organic chemistry, biomedicine and bioconjugation, the term click chemistry has been related to a variety of highly efficient, fast and selective chemical reactions that result in substances and compounds of great importance in the biomedical industry, causing low environmental impact and high chemical yield for the compound of interest. Generally, these reactions originate from the joining of small chemical units containing heteroatoms (C-X-C) [154]. Normally, these reactions occur in the absence of solvents or in the presence of green solvents, such as water, resulting in products with high yields whose byproducts are generally stereospecific, which facilitates their removal from the reaction medium without the need to use extraction techniques that require large amounts of organic solvents during the process. In this case, the purification process often takes place through distillation or crystallization and the isolated products tend to be stable under physiological conditions. Among the main classes of known click reactions are cycloadditions, nucleophilic ring openings, carbonyl chemistry of the non-aldol type and additions to carbon–carbon multiple bonds [155].

**Figure 14:**
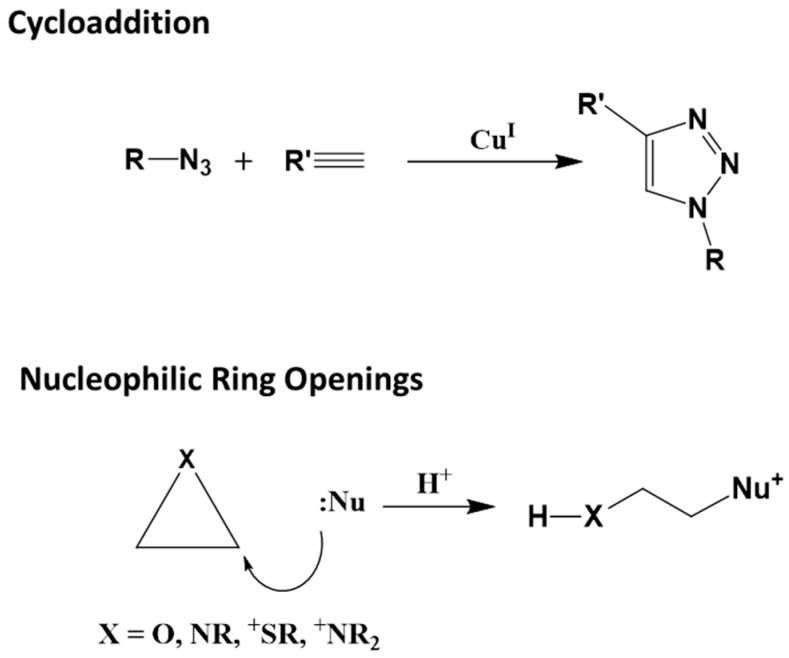
Cycloaddition and nucleophilic ring-opening click chemistry reactions.

Cycloaddition click reactions, whose reactional schemes can be seen in Figure 14, are also known as Huisgen reactions. These are 1,3-dipolar cycloaddition reactions between alkynes and azides catalyzed by copper (I) to produce regioisomers of 1,2,3-triazoles-1,4-disubstituted. Additionally, known as the CuAAC reaction, cycloaddition click reactions also include hetero-Diels–Alder cycloadditions. In nucleophilic ring-opening click reactions, strained heterocyclic electrophiles, such as epoxides, episulfonium ions, aziridines and cyclic sulfates, among others, are opened in the presence of a basic catalyst for the formation of 1,2-difunctionalized compounds. These reactions are highly employed for the post-polymerization modification of polymeric scaffolds, but their reaction byproducts can cause side reactions, affecting the yield and the physical–chemical properties of the products obtained [156].

**Figure 15:**
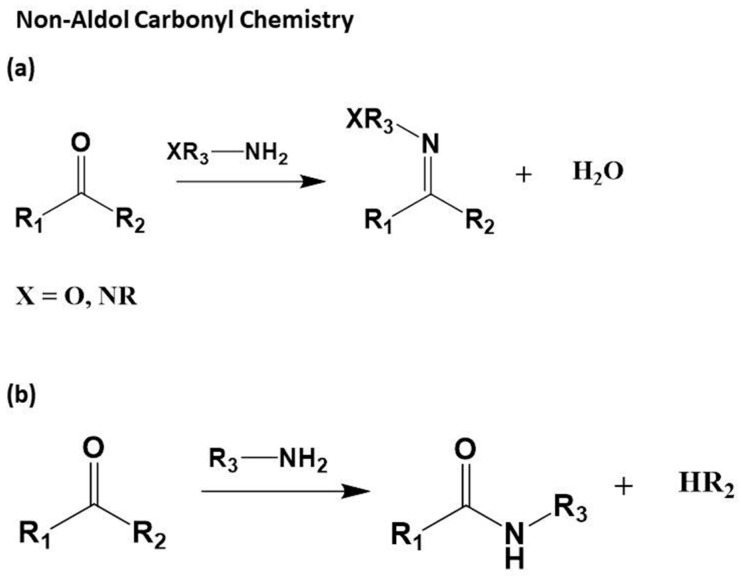
Non-aldol carbonyl click reactions for the (**a**) hydrazone/oxime ether and (**b**) amide/isourea formation.

Non-aldol carbonyl click reactions are reactions that result in the formation of hydrazones, ureas, oximes, amides, thioureas and aromatic heterocycles, among others. Generally, these reactions have high thermodynamic forces, allowing products to be obtained more quickly, which decreases the likelihood of byproduct formation [157]. Examples of reaction schemes of this class can be seen from Figure 15.

**Figure 16:**
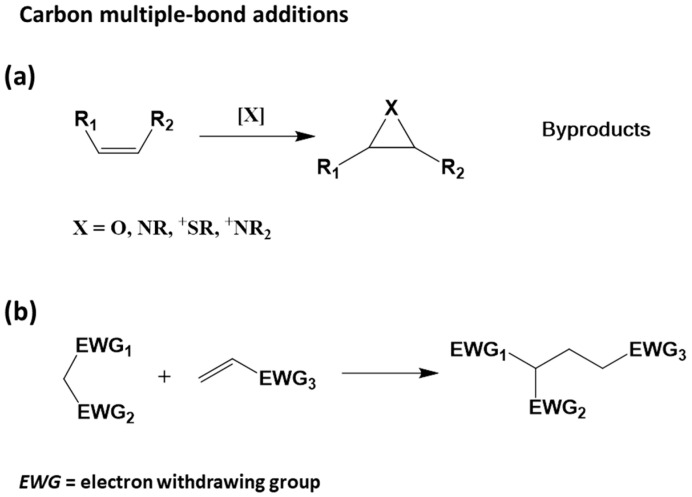
Schematic representations of additions to carbon–carbon multiple-bond click reactions for the formation of (**a**) various three-member rings and (**b**) certain Michael additions.

The additions to carbon–carbon multiple-bond click reactions are mostly represented by Michael reactions, but other examples such as epoxidation, aziridination, nitrosyl halide additions and sulfenyl halide additions also stand out in this class [158]. Some reaction schemes of additions to carbon–carbon multiple-bond click reactions can be seen in Figure 16.

The “click” chemistry approach supports modifications yielding hydrogels suitable for a range of biological applications, including sulfonium functionalization for antibacterial properties [149]. Novel starting materials have expanded the range of hydrogels that may be synthesized using free radical polymerization in aqueous solvent. Wang et al. developed a stable radical-containing self-healing hydrogel by crosslinking acrylic acid and TPYA with *N*,*N*′-methylenebisacrylamide in water. TPYA is an amphiphilic molecule with terpyridine and adamantane motifs that, upon UV irradiation or heating, generates a radical stabilized by the hydrogel network. The hydrogel scaffold may stabilize the radical by reducing oxygen access and promoting charge delocalization by enforcing the π–π stacking of terpyridine groups. The hydrogel demonstrates self-healing behavior in addition to chemical sensing; the disruption of hydrogen bonding and thus hydrogel structure by ammonia led to the loss of the radical and changes in optical properties [159]. In a study linking microscale structure and macroscopic mechanical property characterization, Ihlenburg et al. introduced a novel water-soluble monomer with two methacrylate groups and two quaternary ammonium groups. Though monomer synthesis required organic solvent, thermal free-radical polymerization with sulfobetaine monomers was performed in water at three different monomer:crosslinker ratios, demonstrating the tunability of network nanoscale features and mesh size [160].

Novel hydrogels have also been successfully synthesized in water via controlled radical polymerization (CRP) methods. Theodorou et al. synthesized a brominated maleimide-functionalized CRP initiator that could be coupled to bovine serum albumin (BSA) via thioether linkage or to *β*-galactosidase via amide linkage. The protein-coupled CTA then allowed further polymerization in water to form protein-synthetic polymer conjugates from monomers, including NIPAM. The copper-mediated CRP provides a route to polymer–protein bioconjugate synthesis without organic cosolvents [161]. Additionally, using a CRP technique, in this case photoinduced electron/energy transfer reversible addition–fragmentation chain-transfer (PET-RAFT), Liu et al. developed bilayer hydrogel wound dressings by polymerizing two different monomer formulations in water using visible light, with erythrosin B as a photosensitizer and 2-(dodecylthiocarbonothioylthio) propionic acid (DOPAT) as the chain-transfer agent. The bilayer design addresses the contradictory requirements for wound dressing, specifically, an inner layer that is soft, comfortable, moist and antibacterial, combined with an outer layer that is tough and protective yet elastic. The inner polymer layer consisting of chitosan-*N*-2-hydroxypropyl trimethylammonium chloride-*co*-poly(NIPAM) was synthesized first, with the RAFT process permitting subsequent chain extension to form the outer layer consisting of poly(vinyl alcohol)-*co*-polyacrylamide. Freeze–thaw cycles resulted in PVA crystallization and physical crosslinking to strengthen the outer layer [162]. Wu et al. demonstrated the versatility of PET-RAFT with a novel organic photocatalyst designed based on density functional theory studies. The novel molecule successfully photocatalyzed the RAFT polymerization of multiple monomer types (acrylamides, acrylates and methacrylates) upon green light irradiation at mild pH, even in the presence of oxygen [163]. Further highlighting the versatility of RAFT polymerization in water, Piogé et al. implemented sono-RAFT polymerization-induced self-assembly (PISA) with a macro-RAFT agent synthesized from poly(ethylene glycol) methyl ether acrylate and 2-cyano-5-oxo-5-(prop2-*yn*-1-ylamino)pentan-2-yldodecylcarbonotrithioate (COPYDC) along with NIPAM and BIS. Sono-RAFT-PISA uses sound to create free radicals from water via acoustic cavitation resulting in the formation of local high-temperature pockets [164].

Though not necessarily specifically designed for biomedical applications, the novel materials and synthetic approaches discussed above demonstrate the range of material properties that may be achieved via water-based hydrogel synthesis. For biocompatibility in particular, the use of non-cytotoxic materials such as metal-free catalysis is an important consideration [165]. Montheil introduced a “biocompatible catalysis” approach for bioink formation using aqueous solutions in which silylated hydroxypropylmethyl cellulose crosslinking was catalyzed by NaF and/or glycine [166]. “Catalyst-free” superabsorbent hydrogel synthesis in water was demonstrated by Ahmad et al. via crosslinking *Moringa oleifera* gum and polyvinyl alcohol with borax [167]. As a more biocompatible alternative to the Irgacure 2959 photoinitiator, Bui et al. synthesized a catechol-functionalized hydrogel using dopamine as a photoinitiator to polymerize sulfobetaine methacrylate and BIS in Tris buffer. Dopamine exhibited lower cytotoxicity than Irgacure 2959 and yielded a self-healing, injectable hydrogel [168]. In an innovative green chemistry approach, Fonseca et al. synthesized hydrogels from milk permeate, an agriculture industry waste product, by functionalizing lactose with methacrylic anhydride via ester linkage prior to aqueous UV photopolymerization without requiring a photoinitiator [169].

Toxicity of crosslinkers represents a concern that has been successfully addressed via aqueous polymerization approaches. Aluri et al. successfully demonstrated the use of 1,2-dithane-1-oxide as novel non-cytotoxic crosslinkers for hydrogel formation from either 4-arm PEG thiols or glycosil (thiol-modified hyaluronic acid) in phosphate-buffered saline (PBS). The results indicated that the cyclic thiosulfinate crosslinker exhibited 100-fold lower toxicity in vitro compared to maleimide and vinyl sulfone alternatives [170]. Thongsuksaengcharoen developed a hydrogel-based wound dressing without the use of a toxic chemical crosslinker, catalyst, or organic solvent by covalently linking poly(vinyl alcohol) and citric acid in water in the presence of polyvinylpyrrolidone via microwave irradiation [171].

In addition to biocompatibility, biodegradability is an important consideration for in vivo applications in keeping with green chemistry principles [172]. Wei et al. used VPM as a peptide crosslinker to synthesize PEG maleimide-based hydrogels with PBS as the solvent to serve as a cell scaffold for bone defect repair; the crosslinks could be degraded by a metalloprotease produced by the cells [173]. Though synthetic polymers may support additional tunability and improved mechanical properties, biocompatibility and biodegradability are key advantages of using natural polymers for hydrogel preparation [174,175]. Smith et al. used a dual-oxyamine-modified tobacco etch virus (TEV) peptide to form protease-degradable crosslinks between ketone-modified hyaluron and aldehyde-modified methylcellulose in PBS and media [176]. Additionally, using PBS as a solvent, Upadhyay et al. synthesized a BSA-based hydrogel for drug delivery by employing epichlorhydrin to form ether crosslinks between protein tyrosine hydroxyls [177].

Biocompatibility is an essential requirement of all hydrogels intended for in vivo use, though other properties such as mechanical strength vary based on application. The ability to control and improve upon hydrogel mechanical properties has also been demonstrated using water-based synthesis. Gürdap et al. utilized the temperature sensitivity of certain hydrogels to develop dual-thermoresponsive-core crosslinked micelles with properties suitable for drug delivery. Thermal aqueous RAFT polymerization was used to prepare a poly(sulfobetaine) macroCTA employed in a second aqueous RAFT polymerization step with a crosslinking agent and diethylene glycol methyl ether methacrylate (DEGMA), yielding micelles with a *p*DEGMA crosslinked core and *p*SBMA shell. PSBMA exhibits an upper critical solution temperature (UCST), whereas *p*DEGMA exhibits an LCST, meaning that for drug delivery applications, the *p*SBMA shell would be solvated and hydrophilic, preventing nonspecific adsorption, whereas the *p*DEGMA core would transition to a collapsed state for drug release [178]. Kundu et al. chemically crosslinked carboxymethyl cellulose and xylan with ethylene glycol diglycidyl ether in aqueous NaOH with mild heating to yield a hydrogel supporting the increased loading of vitamin B12 that was able to withstand a greater extent of deformation than either polymer alone [179]. To improve the mechanical strength of gelatin-based hydrogels for tissue engineering, Ma et al. used photoinitiation in aqueous buffer to synthesize a triple-crosslinked hydrogel from methacrylate-functionalized gelatin and alginate. The triple-crosslinked interpenetrating network consisted of the ionic crosslinking of alginate with Ca^2+^, the chemical crosslinking of methacrylate groups, and covalent imine bonds between alginate and gelatin [180].

**Figure 17:**
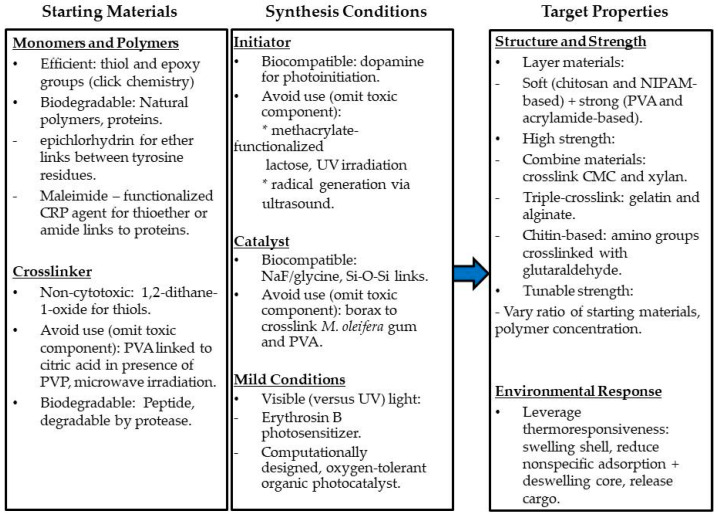
Selected water-based hydrogel synthesis strategies for biological applications. CRP = controlled radical polymerization; PVA = poly(vinyl alcohol); PVP = polyvinylpyrrolidone; CMC = carboxymethyl cellulose.

Demonstrating the tunability of mechanical properties, Bolanta et al. employed an aqueous photoinitiated thiol-acrylate crosslinking of acrylic acid and cysteine-functionalized polyacrylic acid. Varying degrees of swelling and stiffness were achieved based on acrylic acid content and irradiation time, an important feature for versatile in vitro cell growth applications where optimal stiffness depends on cell type [174]. Slawinski et al. utilized photopolymerization with the photoactivation of ruthenium in phosphate buffer to crosslink BSA via the linkage of tyrosine hydroxyl groups in the presence of acetic acid. Tunable stiffness was achieved by adjusting the amount of acetic acid, with decreased acetic acid yielding lower crosslinking density and softer hydrogels suitable for tissue engineering [181]. While hydrogel softness may be advantageous for cell culture and tissue engineering, for applications requiring especially high mechanical strength, Liu et al. described the preparation of ultra-strong reversibly compressible viscoelastic hydrogels using aqueous solutions. Chitin extracted from crab shells was partially deacetylated in aqueous NaOH prior to homogenization, yielding nanofibrils that were subsequently crosslinked using glutaraldehyde via nanochitin amino groups [182]. Figure 17 correlates some of the main starting materials in the synthesis of water based hydrogels, their main synthesis conditions and some biological applications. 

### 6.2. Considerations for Target Applications

In sum, synthesis strategies have been designed by considering the nature of hydrogel–biomolecule interactions as well as the properties of the final hydrogel necessary for the target application. For affinity enrichment and biomarker detection, hydrogels capable of efficient capture and release via fast, simple sample preparation are required [141]. Hydrogels designed for bio-sensing applications must exhibit high sensitivity and selectivity for the molecule of interest in a complex biological matrix [183]. For drug delivery, hydrogels must stabilize and preserve the native structure of the encapsulated biomolecule [184]. Controlling release timing requires that the hydrogel–drug interaction be stable enough to limit burst release while limiting an excessively strong binding interaction that denatures the drug or inhibits release entirely. In the following sections, hydrogels developed for several applications in biochemistry are discussed, with an emphasis on green chemistry and water-based preparation methods.

## 7. Applications in Biochemistry

### 7.1. Drug Delivery

**Figure 18:**
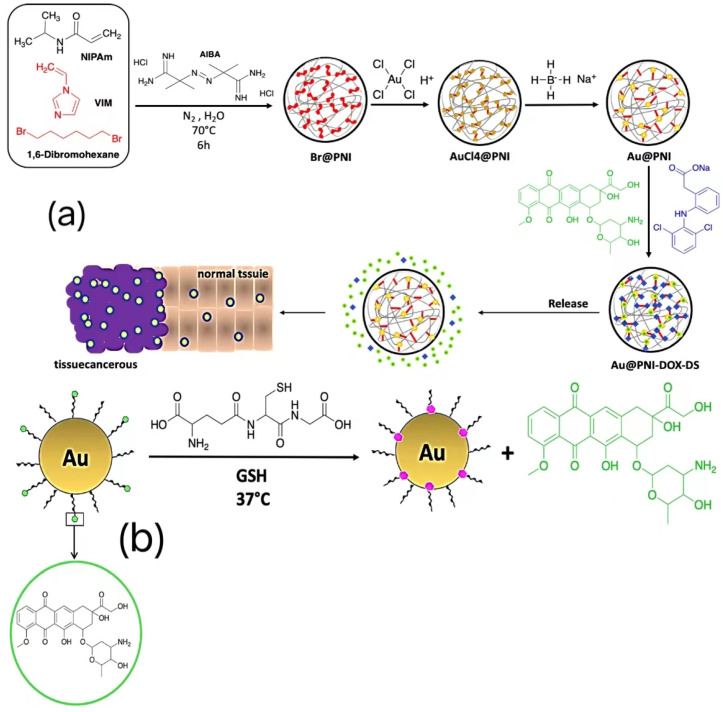
Framework for the (**a**) reaction mechanism developed by Zhou et al. and (**b**) the release profile of doxorubicin (DOX) on the surface of the functionalized gold nanoparticles.

The use of nanogels or hydrogels as a drug delivery carrier has become a powerful tool to overcome the difficulties associated with the low solubility of some drugs and, in turn, their low bioavailability [95]. Nanogels as drug carriers can maintain the dose concentration and protect the drug from the increasing and decreasing effects of temperature and pH in the biological environment [185]. It is imperative to establish productive transporter materials for multi-drug synchronized release profiles to obtain combination therapy [186]. Nanogels have also been thoroughly researched as ideal transports to carry many therapeutics for cancer treatment because of their compact nature [187]. Nanocarriers usually employ four drug-loading mechanisms, including a cavity-loading system, matrix loading, surface loading, and molecular level loading. Zhou et al. used hybrid hydrogels based on NIPAM, 1-vinylimidazole (VIM) and 1,6-dibromohexane in the presence of 2,2′-azobis(2-methylpropionamidine) dihydrochloride (AIBA) embedded in gold nanoparticles as vectors for the simultaneous delivery of doxorubicin, a hydrophobic anticancer drug, and diclofenac sodium, an anionic anti-inflammatory drug, via cation–π interactions and/or electrostatic interactions [188]. The authors observed that in the tumor environment, drug release occurred in a controlled manner through microgel responses to changes in pH (6.8 in the extracellular and 5.0 in the intracellular environment), temperature (37.5 °C) and reduction condition of the medium (presence of glutathione (GSH) as a cellular antioxidant). In addition, in vitro tests showed that drug-loaded microgels could be taken up by cells, allowing the drugs to be released and activated due to the death of cancerous cells without showing toxicity to normal cells. An outline of the reaction mechanism developed by Zhou et al. and the release mechanism of doxorubicin (DOX) on the surface of the functionalized gold nanoparticles can be seen from Figure 18.

### 7.2. Peptide Discovery and Therapeutic Development

The emergence of multidrug-resistant bacteria and the ongoing threat of viral pathogens has inspired new strategies for therapeutic discovery [189]. The natural immune defenses deployed by diverse species such as *Alligator mississippiensis* and *Varanus komodoensis* to survive in pathogenically challenging environments may inform the development of novel antibacterial and antiviral therapeutics [190,191]. However, the identification of host-defense peptides (HDPs) in non-model organisms is complicated by a lack of well-curated sequence information. The primary sequence and structural diversity of HDPs, even among closely related organisms, further complicates HDP discovery via bioinformatics approaches.

**Figure 19:**
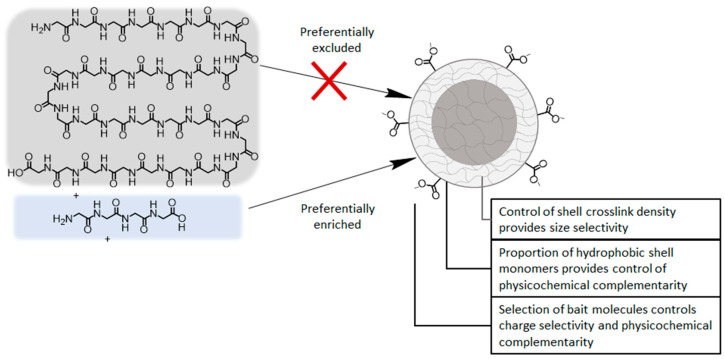
Concept of core–shell particle harvest. Preferential enrichment of target biomolecules, in this case, amphipathic and cationic peptides, is achieved with a particle design that provides size selectivity and physicochemical complementarity to targets.

Considering the cationic and amphipathic nature of many known HDPs, Bishop et al. developed hydrogel microparticles with hydrophobic moieties and anionic affinity baits to enrich low-abundance candidate HDPs from small-volume plasma samples. Poly-NIPAM-based particles were synthesized via aqueous one-pot free radical precipitation polymerization with BIS as the crosslinker and AA and 2-acrylamido-2-methylpropane sulfonic acid (AMPS) as baits. A second particle design consisted of a *p*NIPAM-*co*-AA core with a *p*NIPAM shell [192]. In addition to targeting low-abundance peptides via hydrophobic and electrostatic interactions, particles were designed to provide the size exclusion of larger proteins via the crosslinked polymer network (Figure 19) [193]. A 1:1 mixture of the two particle types were used to capture and enrich peptides from 100 μL of *Alligator mississippiensis* plasma, resulting in the identification of 568 peptide sequences via database search and the de novo sequencing of high-resolution MS/MS spectra [194]. Based on peptide mass, charge, and predicted activity, eight peptides were selected for synthesis and testing against a panel of Gram-negative and Gram-positive bacteria: *Escherichia coli* (ATCC 25922), *Bacillus cereus* (ATCC 11778), *Pseudomonas aeruginosa* (ATCC 9027), and *Staphylococcus aureus* (ATCC 25923). The results suggest that peptides APOC1_64–68_ and APOC1_67–88_, both nested fragments of apolipoprotein C1, as well as A1P_394–428_, a fragment of alpha-1 anti-proteinase, exhibit broad-spectrum antimicrobial activity, with EC50 within 2 log of LL37 for the *Escherichia coli*, *Bacillus cereus*, and *Pseudomonas aeruginosa* strains tested. Though A1P_394–428_ was also effective against *S. aureus*, with EC_50_ within 2 log of LL37, APOC1_64–68_ and APOC1_67–88_ were less effective against this bacteria strain [192,194].

Barksdale et al. performed further studies of peptide activity against clinically isolated, multidrug-resistant strains of *Staphylococcus aureus*, *Escherichia coli*, *Pseudomonas aeruginosa*, and *Acinetobacter baumannii* [195]. APOC1_64–68_ and APOC1_67–88_ exhibited strong (EC_50_ < 5 ug/mL) activity against *S. aureus*, and very strong (EC_50_ < 1 ug/mL) activity against *A. baumannii*, methicillin-resistant *S. aureus*, and multidrug-resistant *P. aeruginosa*, though reduced effectiveness against *E. coli* was observed compared to previous work [192]. A1P_394–428_ exhibited only weak effectiveness against *P. aeruginosa* and was not effective against multidrug-resistant *P. aeruginosa*. However, this peptide exhibited increased effectiveness against multidrug-resistant strains of *E. coli*, *S. aureus*, and *A. bauminnii* compared to antibiotic-sensitive strains. Taken together, these results suggest that the selected peptides identified from the hydrogel particle bioprospecting process may exhibit broad-spectrum effectiveness against multidrug-resistant strains at concentrations that are not cytotoxic nor hemolytic to host cells [195].

To evaluate the versatility of the particle design for candidate HDP discovery, particles were used to enrich peptides from Komodo dragon (*Varanus komodoensis*) plasma, resulting in the identification of 48 potential candidate antimicrobial peptides. Employing a similar strategy to the *A. mississippiensis* study, eight histone-derived peptides were selected for synthesis and evaluation based on mass, charge, and predicted activity. Seven of the eight peptides exhibited effectiveness against *S. aureus* and *P. aeruginosa* at EC_50_ values comparable to LL37, whereas the remaining peptide was only effective against *P. aeruginosa* [141]. Building on these results, the synthetic peptide DRGN-1 was designed by reversing the *N*-terminal serine and proline residues of VK25, one of the eight *V. komodoensis* peptides which exhibited activity against both bacterial strains tested. Further evaluation of peptide structure and activity suggested that DRGN-1 exhibited superior antimicrobial effectiveness compared to VK25. In addition, unlike VK25, DRGN-1 promoted the healing of a biofilm-infected wound in a BALB/c mouse model, with complete healing occurring more quickly than with LL37 treatment. It was hypothesized that the superior activity of DRGN-1 may be attributed to enhanced stability, since the *N*-terminal Ser residue of VK25 may target this peptide for degradation via the bacterial ClpXP system [196]. These results indicate that strategies to synthetically tailor hydrogel particle architecture and functionality to complement target molecules, when combined with analysis of the identified peptide sequences, may provide promising avenues for therapeutic development [197].

To expedite sample processing and optimize peptide capture, additional particle formulations were evaluated [142]. The hydrophobic monomer *N*-*tert*-butylacrylamide (*t*BA) was introduced to stabilize the polymer backbone [198]. BIS was used as a chemical crosslinker in the aqueous free-radical precipitation polymerization syntheses. Specifically, particles with a *p*NIPAM-*co*-*t*BA-*co*-methacrylate core and NIPAM shell were prepared and subsequently saponified to introduce acidic functionalities to the methacrylate groups. In addition, particles with a *p*NIPAM-*co*-*t*BA core and *p*AA shell, as well as particles incorporating AMPS (*p*NIPAM-*co*-*t*BA-*co*-AMPS-*co*-AA) were synthesized [199]. Particles were characterized via DLS and SEM. The incorporation of *t*BA stabilized particles against hydrodynamic diameter decrease at higher temperatures, a property desirable for consistency in harvest performance, though greater thermoresponsiveness was observed for particles incorporating both AA and AMPS acidic monomers. The use of *t*BA increased the proportion of unique peptides identified from alligator plasma with a net charge from +5 to +8, which was attributed to decreased polymer flexibility and thus an enhanced exclusion of larger proteins [142]. Since limited overlap was observed between sets of peptides captured in seven experimental replicates, techniques permitting greater synthetic control than is observed with free-radical precipitation polymerization may improve harvesting consistency.

As with HDPs, snake venom toxins exhibit diverse primary sequences, which complicates the development of broad-spectrum antivenom. Shea et al. implemented a novel approach to antivenom development by considering the common structural scaffolds exhibited by classes of venom peptides and engineering a nanoparticle formulation for the broad-spectrum targeting of venom toxins [200]. Monodisperse hydrogel nanoparticles with a monomer formulation of 20% AA, 40% *N*-phenylacrylamide, 25% NIPAM, and 15% BIS were synthesized via aqueous precipitation polymerization (10% acetone solution in water). Particles selectively sequestered low-molecular-weight (<15 kDa) venom toxins from a complex serum matrix. Not only did particles selectively capture venom peptides, but the administration of nanoparticles after venom injection significantly reduced dermonecrosis in a mouse model [201]. Though the particle inhibition of in vivo venom toxicity decreased with time from venom exposure, the lack of particle cytotoxicity and the effectiveness of venom sequestration provide further support for the suitability of hydrogels in the selective binding of target classes of biomolecules [202].

### 7.3. Biomarker Detection

The sensitive detection of low-abundance biomarkers such as peptides, metabolites, and viral proteins is a challenging yet essential task for early disease diagnosis. Nanoparticles such as *p*NIPAM-based core–shell particles have been used in proteomics studies to address the challenge of isolating low-abundance, low-molecular-weight biomarkers from serum and plasma. Advantages of NIPAM for this application include high colloidal stability and batch-to-batch reproducibility. Vinylsulfonic acid (VSA) coating inhibits the particle uptake of albumin fragments below the molecular weight cutoff of the polymer network [203]. Biomolecule selectivity may be customized by changing the identity of the bait molecule. For example, Fredolini et al. used Cibacron Blue as an affinity bait to enrich low-abundance proteins from human serum in a shotgun proteomics study that identified 24 invasive ductal carcinoma candidate biomarkers. Particles were synthesized via aqueous precipitation polymerization and were functionalized with a vinylsulfonic acid (VSA) shell [204]. Culver et al. combined NIPAM-based hydrogels with gold nanomaterials in a biosensing system for tear biomarkers [205]. The localized surface plasmon resonance (LSPR) wavelength of gold nanomaterials is sensitive to the refractive index, which increases significantly upon protein binding to *p*NIPAM. Silica gold nanoshells were surface-modified with a *p*NIPAM-*co*-methacrylic acid hydrogel shell via aqueous precipitation polymerization and characterized with TEM. Hydrogel shells were not thin or uniform, but exhibited pH-dependent swelling behavior. Increasing the solution’s pH from 5.5 to 7.4 corresponded to increased swelling and a greater capacity for lactoferrin and lysozyme. The selectivity for lactoferrin and lysozyme was attributed to favorable electrostatic interactions between these high-pI proteins and the hydrogel and the elevated concentration of these proteins in tears.

Combining the strategies of NIPAM-based particles for biomarker capture with targeted drug delivery, Fruehauf et al. developed nanoparticles responsive to lactic acid, a metabolite present in the hypoxic environment near tumors [147]. The monomer formulation consisted of NIPAM, selected for its low affinity for plasma proteins, and an oxamate derivative [112]. Particles were synthesized via aqueous precipitation polymerization with 2 mol% BIS as a crosslinker to achieve size uniformity without restricting swelling capacity [206]. Lactate dehydrogenase binds oxamate moieties in the particle to create a noncovalent crosslink network. Particle design takes advantage of the natural structural compatibility between enzymes and substrates by linking particle swelling behavior to elevated concentrations of lactic acid, which displaces oxamate and disrupts the noncovalent crosslink network, leading to particle swelling [147]. Shigemitsu et al. also designed a hydrogel programmed to swell and release sequestered protein based on in vivo protein–protein interactions [146]. The hydrogel was designed to respond to non-enzymatic protein binding via enzyme-activity triggers (EAT) consisting of biotin-linked benzenesulfonamide, a carbonic anhydrase inhibitor [207]. Anhydrase and EAT were mixed with agarose and diphenylalanine derivatives in aqueous buffer (HEPES) with hydrophobic *N*-terminal groups to form a peptide-based supramolecular hydrogel. Confocal laser scanning microscopy indicated that agarose, incorporated to enhance mechanical toughness, was entangled but minimally interacted with the dipeptide component. Avidin binding to the EAT biotin moiety resulted in inhibitor dissociation from the enzyme. The activated enzyme cleaved the hydrophobic peptide *N*-terminal groups, increasing hydrogel hydrophilicity and swelling [146].

Targeted biomarker detection has also been achieved by applying azide–alkyne cycloaddition for hydrogel synthesis or hydrogel functionalization. Al Sulaiman et al. attached alkyne-functionalized peptide nucleic acid probes to azido-functionalized alginate crosslinked with calcium chloride via copper-catalyzed azide–alkyne cycloaddition in aqueous buffer [208]. The hydrogel was coated on microneedles via electrostatic interactions. The high swelling capacity and porosity of hydrogels was combined with the high microneedle surface area for the sensitive, noninvasive detection of nucleic acid biomarkers. A different approach for bait incorporation is the use of thiol-reactive groups. Roh et al. employed thiol chemistry to functionalize PEG-based hydrogels for protein detection [136]. Hydrogels were synthesized from an aqueous prepolymer solution of PEGDA 700 and PEG 600 via stop-flow lithography with photo masks to achieve geometry complementary to protein targets. Since stop-flow lithography achieved low monomer conversion, residual alkene groups were coupled to antibodies via a heterobifunctional thiol PEG linker. The results suggested that sensitivity comparable to the ELISA assay was achieved as a result of post-functionalization leading to a higher density of antibody conjugation. In a different study, Al Sulaiman et al. also employed photolithography with masks to synthesize PEGDA-based hydrogels with controlled shape and size, though miRNA probes were covalently incorporated in situ. Hydrogels were prepared via the UV irradiation of fibrous substrates that had been incubated with a prepolymer solution consisting of PEGDA, PEG, and miRNA probes in Tris-EDTA. The limit of detection was estimated as 50 fM, with the level of sensitivity attributed to the ability to create a high-resolution, small-scale patterning of hydrogels within a rigid glass matrix [134].

In addition to stop-flow lithography photomasks, molecular imprinting provides a means of controlling hydrogel geometry for enhanced biomolecule selectivity. Chen et al. developed a molecularly imprinted hydrogel coating via polymerization and crosslinking of acrylamide (AM) in the presence of bovine serum albumin (BSA) in aqueous solution. Compared to uncoated silver nanoparticles, the molecularly imprinted hydrogel coating improved the surface-enhanced Raman scattering (SERS) detection of BSA by two orders of magnitude [137]. Ying et al. also studied the effect of molecular imprinting on the extent of BSA adsorption to the final hydrogel matrix. NIPAM, AM, and AMPS were polymerized and crosslinked with BIS in the presence and absence of BSA via aqueous free-radical polymerization [131]. Imprinted polymers exhibited larger, less uniform pore sizes and a relatively higher level of BSA binding at 15 weight% crosslinking. However, differences in binding between imprinted and non-imprinted polymers were less pronounced for lower or higher degrees of crosslinking, suggesting the necessity of achieving a crosslinking density low enough to permit biomolecule transport but high enough to stabilize imprinting sites. Hayakawa et al. applied molecular imprinting to address the problem of reducing the cellular uptake of nanoparticles in vivo [138]. NIPAM was crosslinked with pyrrolidyl acrylate and 2-methacryloyloxyethyl phosphorylcholine via emulsifier-free precipitation polymerization in the presence of human serum albumin with PBS as the solvent. Compared to non-imprinted particles, reduced cellular uptake was observed for particles with albumin recognition capability.

An alternative approach for biomolecule detection involves host–guest interactions. For example, Yan et al. employed supramolecular *β*-cyclodextrin-based hydrogels for chiral amino acid separation [209]. Positively charged copper-modified *β*-cyclodextrin was combined with negatively charged laponite clay in water to form a supramolecular hydrogel via electrostatic interactions. SEM characterization of the hydrogel indicated the chiral cyclodextrin cavity was unoccupied. Binding studies using UV-Vis spectroscopy indicated the hydrogel exhibited a greater extent of *L*-tryptophan binding as compared to *D*-tryptophan, as well as other *L*-amino acids present in a mixture.

### 7.4. Nanogels as an Imaging Probe

A powerful early-stage screening technique for cancer is tumor imaging [210]. Kim et al. reported an injectable magnetic resonance imaging (MRI)-monitored long-term medicinal hydrogel (MLTH) for the recognition and localization of brain tumor tissues [211]. This biocompatible magnetic poly(organophosphazene) hydrogel could theoretically be a compostable imaging medium for anticancer drugs, which would be simpler to operate relative to the implantable Gliadel Wafer device containing the chemotherapy drug carmustine. According to the authors, MLTH can illustrate the escape and dissemination of nanomagnets into tissues, which provides important evidence to measure the volume of the drug that reaches the tumor tissue. Traditional MRIs cannot detect drug diffusion inside a tissue. Pertinently, this approach could assess medication effectiveness, which varies from person to person. MLTH helps doctors to evaluate the quantity and potency of the disseminated medication. This approach will facilitate tailored treatment by making appropriate improvements in the dose or medication.

Lux et al. used positron emission tomography (PET) for the visualization of primary tumors and metastases using nanogels containing metal-chelating crosslinkers [212]. The authors tested the chelation stability of nanogels containing diethylenetriaminepentaacetic acid (DTPA)-based crosslinkers, 1,4,7,10-tetraazacyclododecane-1,4,7,10-tetraacetic acid (DOTA) and 1,4,7-triazacyclononane-1,4,7-triacetic acid crosslinker (NOTA). Mouse serum tests showed that ^64^Cu was held quite stable by NOTA-based nanogels; thus, they were used for the in vivo PET/CT scanning of tumor-bearing mice along with DOTA-based nanogels. The authors observed that ^64^Cu-NOTA-crosslinked nanogels prompted their better aggregation in the tumor through the improved permeation and retention (EPR) effect, as well as lower liver signal, as opposed to ^64^Cu-DOTA-crosslinked nanogels, further supporting their improved stability of chelation. Bioavailability tests also indicated a higher concentration of ^64^Cu in tumors for NOTA nanogel administration versus DOTA nanogels (16.6% vs. 12.9% injected dose per gram at 48 h). ^64^Cu-NOTA-crosslinked nanogels also identified metastases with greater absorption than in subcutaneous tumors in certain situations. The strong chelation stability of these nanogels, high passive tumor uptake, long blood circulation time, and relatively low liver and spleen uptake indicate their suitability for molecular imaging and drug delivery, for example.

## 8. Perspectives

Novel synthetic strategies and thorough computational and experimental studies of hydrogel–biomolecule interactions in model systems have diversified and advanced the development of hydrogel systems for biochemistry applications. For example, though hydrogel particles synthesized via free-radical precipitation polymerization performed well for the enrichment of low-abundance cationic peptides, the limited reproducibility of the results may arise in part from the polydispersity of the polymer product achieved in free radical polymerization [142]. The application of techniques providing greater synthetic control, such as RAFT, provide a means of better controlling batch-to-batch reproducibility needed for consistent performance in the target application [213]. The remaining synthetic challenges include the ability to exert greater structural control over the final hydrogel and large-scale synthesis of hydrogels, exhibiting the multilayer architecture and monomer sequence control exhibited by biopolymers [214,215]. Continued improvements in methodology to incorporate diverse functional groups, especially at larger scale, would be especially relevant to improving multiplexing capabilities of biosensing platforms [208,215]. Furthermore, novel particle functionalization approaches that select for biomolecules involved in specific in vivo interactions, such as host-defense peptides, may expedite discovery and characterization efforts, with broader relevance to antiviral therapeutic development.

Model systems have provided important insight into correlations between synthetic parameters, hydrogel properties, and biomolecule interactions. Building on this work, larger-scale experimental studies to quantify the efficiency of biomarker enrichment may establish more systematic links between hydrogel properties and performance [128]. The quantitative validation of the selectivity and specificity of hydrogel–biomolecule interactions in more complex matrices is especially important for intended clinical applications. Though high selectivity may be observed in a model system, such systems do not account for the fact that in more complex matrices, biomolecules tend to form a shell layer on the hydrogel surface, which alters hydrogel surface charge properties [122]. Such systematic studies would also provide insight into the in vivo performance of hydrogels intended for drug delivery, since in vivo release kinetics of encapsulated proteins may be affected by proteins in the surrounding matrix [126]. In addition, quantitative binding data would inform the fine-tuning of particle properties to achieve biomolecule interactions that are strong enough to retain selectivity in the presence of interfering, high-abundance proteins, yet moderate enough to permit the near-complete recovery of the biomarkers of interest.

## 9. Conclusions

The use of hydrogels, nanogels or microgels in the biomedical field has become a useful tool both in the capture of macro and micromolecules of pharmaceutical interest, as well as in the synthesis of functionalized imaging probes and in the controlled release of drugs and bioactives. Throughout this review, the different classifications of hydrogels, their physical–chemical characteristics and the different technologies adopted for the synthesis and modification of these materials were discussed. In addition, innovative applications of nanogels related to both the capture of antimicrobial peptides as well as their functionalization with inorganic nanoparticles for controlled drug release and as biomarkers were discussed. The development of new techniques and functionalized devices based on hydrogels has also been proposed as a powerful alternative with low toxicity and high biodegradability when compared to drug carriers and bioactive agents currently used in the biomedical field. Finally, green chemical routes were proposed for the synthesis of hydrogels from natural bio-based monomers or biopolymers, thus allowing the obtainment of biodegradable, biocompatible and low-cost biomaterials.

## Figures and Tables

**Table 1:** Examples of natural bio-based monomers, their most used copolymers and main applications in the biomedical field.

Natural Bio-Based Monomers	Polymer-Based Hydrogel	Applications
Chitosan (CS)	- Chitosan and dextran; - Chitosan/disulfiram (Ch/DSF) hydrogel.	- Topical drug delivery for wound healing [30]; - Anticancer drug carrier [31].
Glycolic acid (GA)	- Chitosan/PLGA; - Poly (lactic-co-glycolic) acid (PLGA).	- Encapsulation of an enriched flavonoid fraction of *Cecropia glaziovii* Snethl [32]; - Biomimetic injectable microspheres through inclusion of bioactive nanoparticulates [33].
Lactic acid (LA)	- PLA-CS; - PLA-polyurethanes.	- pH-responsive hydrogels for drug delivery [34]; - Thermosensitive hydrogels for drug delivery [35].
Ethylene glycol (EG)	- PEG-succinic acid (SA); - PEG hydrogels crosslinked by Poloxamer 407; - Deformable propylene glycol-containing liposomes hydrogel.	- Elastic nanospheres for biological scaffolds [36]; - Injectable hydrogel for drug delivery [37]; - Vaginal drug delivery system [38].
Propylene oxide (PO)	- PEO–PPO; - PPO /PEO-cellulose hybrid nanocomposite hydrogel.	- Transdermal drug delivery [39]; - Drug delivery [40].
Collagen	- Bacterial cellulose/collagen hydrogel; - Alginate microsphere–collagen composite hydrogel.	- Wound healing [41]; - Ocular drug delivery and implantation [42].
Fibrin	- Platelet-rich fibrin in alginate–hyaluronic acid hydrogel; - Injectable fibrin hydrogels.	- Vascularized substitution for myocardial infarction [43]; - Cardiac stem cell therapy and tissue engineering [44].
Fibrinogen	- Decellularized skin extracellular matrix (dsECM) in fibrinogen hydrogel; - Bifunctional aptamer–fibrinogen macromer.	- Skin bioprinting applications [45]; - VEGF delivery and skin wound healing [46].
Alginate	- Magnetic nanocellulose alginate hydrogel beads; - Alginate/magnetic polycaprolactone (PCL).	- Drug delivery [47]; - Nerve tissue engineering [48].
Gelatin	- Alginate/gelatin/carbon nanofibers; - Gelatin hydrogel microspheres (GM) containing pifithrin-α (PFT) of a p53 inhibitor (GM-PFT).	- Electrically conductive scaffolds for tissue engineering [49]; - Anticancer drug [50].
Hyaluronic acid	- PNIPAM/hyaluronic acid; - Polydopamine crosslinked thiol-functionalized hyaluronic acid hydrogel.	- Cartilage tissue engineering [51]; - Angiogenic drug delivery [52].

**Table 2:** Crosslinking reactions for the synthesis of nanogels from polymeric precursors.

Reactions	Characteristics	Applications
Disulfide crosslinking	- Nanogels with great potential as carriers for controlled drug delivery scaffolds to target specific cells. - Prepared by self-crosslinking amphiphilic random copolymers (PEG hydrophilic unit and pyridyl disulfide hydrophobic and crosslinkable unit). - Form nanoscale assemblies in aqueous solutions. - Disulfide can reversibly undergo reduction to thiols depending on the environmental thiol concentration. - The thiol groups can react with reactive groups such as activated disulfides, maleimides, iodoacetyl groups, and some thiol-containing biomolecules at pH > 8. - The products need to be purified to remove unreacted crosslinking agents and byproducts.	Intracellular delivery of chemotherapeutic agents [77]. Nanocarrier for intracellular protein delivery [78].
Amide crosslinking	- Reactive with carboxylic acids, activated esters, isocyanates, iodides, among others. - Supramolecular assembly of amphiphilic block copolymers into micelles, followed by covalent crosslinking throughout the shell layer. - Forms shell-crosslinked knedel-like structures (SCKs). - Developed by the Wooley group, the particles exhibit an adjustable crosslinking degree. - Main polymer: poly(acrylic acid), used as hydrophilic component and a crosslinkable block. - The products need to be purified to remove unreacted crosslinking agents and byproducts.	Multifunctional imaging [79]. Therapeutic agents and targeted biomedical applications [80].
Imine crosslinking	- Covalent imine bonds used to crosslink linear polymer chains into spherical crosslinked nanogels and core crosslinked star (CCS) polymers (Schiff-base reaction). - Reaction occurs in mild conditions (no catalyst needed). Groups: aldehyde and amine or hydrazide. - The products need to be purified to remove unreacted crosslinking agents and byproducts.	Polymer–protein conjugates [81]. Drug or fragrance delivery [82].
Copper-free click chemistry crosslinking	- Bio-orthogonal click reactions that do not require the presence of a copper catalyst (cytotoxic). - Usually occurs in alkyl units with amino groups immobilized in the particle shell via amidation of the hydrophilic polymeric micelle. - pH-dependent reaction speed. - High specificity; tolerant of a wide variety of functional groups; applicable under mild reaction conditions. - The products need to be purified to remove unreacted crosslinking agents and byproducts.	Cell and protein/peptide encapsulation [83,84]. Drug delivery [85]. Dye separation [86].
Photo-induced crosslinking	- Used to stabilize assemblies of polymers that are functionalized with dimerizable or polymerizable units. - Purification steps are not necessary (clean method); however, it shows cytotoxicity issues. - Most common reactive groups: coumarin or alkene. - Most common sources of polymerization: UV irradiation and photo initiator.	Drug delivery [87,88].

## Data Availability

Data sharing is not applicable to this article as no new data were created or analyzed in this study.
